# Intrafamily and intragenomic conflicts in human warfare

**DOI:** 10.1098/rspb.2016.2699

**Published:** 2017-02-22

**Authors:** Alberto J. C. Micheletti, Graeme D. Ruxton, Andy Gardner

**Affiliations:** School of Biology, University of St Andrews, Dyers Brae, St Andrews KY16 9TH, UK

**Keywords:** war, sex-biased dispersal, parent–offspring conflict, sexual conflict, intragenomic conflict, genomic imprinting

## Abstract

Recent years have seen an explosion of multidisciplinary interest in ancient human warfare. Theory has emphasized a key role for kin-selected cooperation, modulated by sex-specific demography, in explaining intergroup violence. However, conflicts of interest remain a relatively underexplored factor in the evolutionary-ecological study of warfare, with little consideration given to which parties influence the decision to go to war and how their motivations may differ. We develop a mathematical model to investigate the interplay between sex-specific demography and human warfare, showing that: the ecology of warfare drives the evolution of sex-biased dispersal; sex-biased dispersal modulates intrafamily and intragenomic conflicts in relation to warfare; intragenomic conflict drives parent-of-origin-specific patterns of gene expression—i.e. ‘genomic imprinting’—in relation to warfare phenotypes; and an ecological perspective of conflicts at the levels of the gene, individual, and social group yields novel predictions as to pathologies associated with mutations and epimutations at loci underpinning human violence.

## 1. Introduction

Recent years have seen an explosion of interest in ancient human warfare [1–18]. Discoveries of prehistoric mass graves and other striking evidence of lethal intergroup conflict have challenged a traditional view that our ancestors were relatively peaceful [19–22] and have spurred strong multidisciplinary effort into understanding the incentives for human intergroup violence [4,9,13,18,23–25]. Although quantitative theoretical progress on this topic has been relatively slow, analysis of mathematical models has yielded a number of important insights into the evolutionary and ecological drivers of war. In particular, Lehmann & Feldman's [7] study of the evolution of belligerence and bravery behaviours, in the context of a population model with sex-specific demography, has highlighted a possible key role for kin selection in incentivizing adolescent males to altruistically cooperate in warfare—paying personal costs, but yielding benefits, such as additional resources or mating opportunities, for their groupmates—even in the context of large groups (where average within-group relatedness is low).

Paradoxically, conflicts of interest remain a relatively neglected factor in the evolutionary-ecological study of human intergroup violence, with little consideration given to which parties influence the decision to go to war and how these various parties' interests might differ. For instance, while Lehmann & Feldman [7] assumed that each adolescent male's behaviour is determined by his father's genotype, such that it is the inclusive-fitness interests of the father that govern the son's belligerence and bravery in relation to warfare, the son's own interests are liable to be different from his father's, especially in relation to selfless acts that may benefit his siblings but incur a severe personal cost. This suggests the potential for parent–offspring conflict (*sensu* [26]). Moreover, the interests of the individual's mother are also liable to differ from those of the father, owing to sex-specific demographic factors—such as sex-biased dispersal—that are expected to generate sex differences in relatedness to groupmates, suggesting the possibility for sexual conflict (*sensu* [27]). Furthermore, sex-specific demographic processes have been shown to drive conflicts of interest between an individual's maternal-origin and paternal-origin genes with respect to social behaviour [28–33], and accordingly there may even be intragenomic conflict (*sensu* [34]), of a form that has been implicated in the evolution of parent-of-origin-specific gene expression, or ‘genomic imprinting’ [35–37]. This renders individuals vulnerable to a range of debilitating cognitive, behavioural and growth disorders [38], some of which have been linked with aggression and violence [39–41]. However, such conflicts of interest remain underexplored.

Here, we determine the scope for—and consequences of—parent–offspring conflict, sexual conflict, and intragenomic conflict in relation to warfare. We reformulate and generalize Lehmann & Feldman's [7] model to consider control of belligerence and bravery by either the adolescent male, his mother, his father, his maternal-origin genes, or his paternal-origin genes. We use this extended model to investigate: (i) the evolution of sex-specific dispersal in the context of the ecology of warfare, (ii) how sex-biased dispersal modulates intrafamily and intragenomic conflicts in relation to warfare, (iii) how intragenomic conflicts of interest can drive genomic imprinting [35], and (iv) the phenotypic and pathological consequences of different classes of mutation and epimutation at imprinted loci underpinning intergroup violence phenotypes.

## 2. Material and methods

Following Lehmann & Feldman [7], we consider a large population separated into groups of *N*_f_ adult females and *N*_m_ adult males, connected by random migration. At the beginning of the life cycle, each adult female produces a large number *K*_f_ of daughters and a large number *K*_m_ of sons, then dies, and her offspring mature to become subadults. Each subadult disperses away from their natal group with probability *d*_f_ for females and *d*_m_ for males, and each disperser dies in the process with probability *λ*_f_ for females and *λ*_m_ for males, with survivors arriving at random groups. Accordingly, following dispersal, the probability that an individual is a migrant is 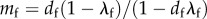 for females and 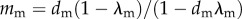 for males. In every generation, each post-dispersal group is in a position to attack one randomly chosen group—which it does with probability *a*(*A*_att_), where *A*_att_ is the average level of belligerence exhibited by subadult males in the attacking group and 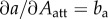 is the marginal increase in the probability of the group attacking another—and to be attacked by one other group. If war is initiated, the attacking group wins with probability 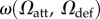, where *Ω*_att_ and *Ω*_def_ are the average levels of bravery exhibited by subadult males in the attacking and defending groups, respectively, and where 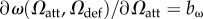 is the marginal increase in the probability of the attackers winning the war (for simplicity, we assume that bravery is equally important in defence: 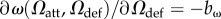). Following warfare: in non-attacked groups, individuals compete for breeding positions against their same-sex groupmates, each subadult male having competitiveness *t*(*A*_ind_), where *A*_ind_ is his level of belligerence and 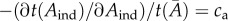 is the competitive cost of belligerence; in groups that successfully defend themselves from attack, individuals compete for breeding positions against their same-sex groupmates, each subadult male having competitiveness 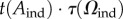, where *Ω*_ind_ is his level of bravery and 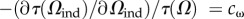 is the competitive cost of bravery; and in conquered groups, individuals compete for breeding positions against their same-sex groupmates and their same-sex attackers, each subadult male having competitiveness 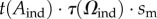 if they belong to the defeated group and 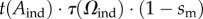 if they belong to the conquering group, and each subadult female having a competitiveness *s*_f_ if they belong to the defeated group and 1 − *s*_f_ if they belong to the conquering group. That is, while a male's belligerence phenotype is always expressed and always incurs a competitive cost, his bravery phenotype is only expressed and only incurs a competitive cost when his group attacks or is attacked by another group. We perform a kin-selection analysis [42–48] to determine how selection acts upon female dispersal, male dispersal, belligerence, and bravery (see the electronic supplementary material for details).

## 3. Results

### (a) Sex-biased dispersal

**Figure 1.**
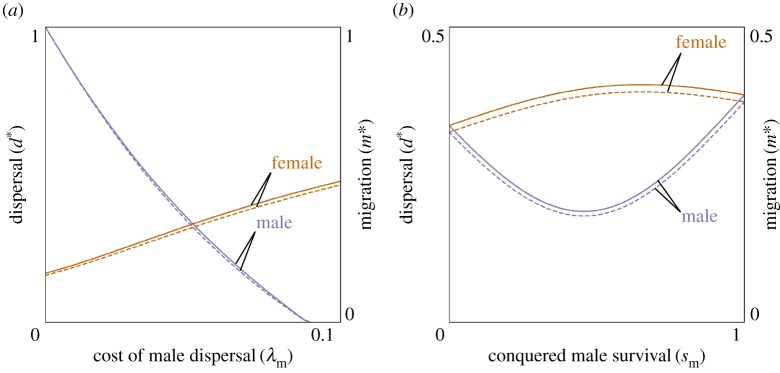
Evolution of sex-biased dispersal and migration. Convergence-stable levels of female dispersal (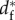, solid orange line), male dispersal (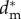, solid purple line), female migration (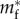, dashed orange line), and male migration (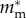, dashed purple line) as functions of cost of male dispersal (*λ*_m_; (*a*); other parameter values are *λ*_f_ = 0.05, *s*_f_ = 1, *s*_m_ = 0, *N*_f_ = *N*_m_ = 10, 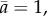 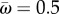) and the probability that a conquered male obtains a breeding spot (*s*_m_; (*b*); other parameter values are *λ*_f_ = *λ*_m_ = 0.05, *s*_f_ = 1, *N*_f_ = *N*_m_ = 10, 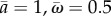). (Online version in colour.)

Sex-biased dispersal is observed in many taxa and, on the basis of population genetic data [49] and dispersal patterns of African apes [50] and modern hunter–gatherers [51], it is understood that female-biased dispersal (patrilocality) was the ancestral condition for humans [29]. However, the causes of these patterns remain unclear and are much debated: theoretical work has identified possible drivers of sex-biased dispersal in mating systems, inbreeding avoidance and competition and cooperation between kin (reviewed in [50]; see also [52]) and many anthropologists have focused on the greater importance of kin recognition and associated cooperation between male kin to explain patrilocality (reviewed in [53]). Here, we investigate the evolution of sex-specific dispersal in a population undergoing recurrent acts of war. Predictably, we find that sex differences in the mortality cost of dispersal can drive sex-biased dispersal (figure 1*a*). More surprisingly, we find that the ecology of warfare itself [15] can drive the evolution of sex-biased dispersal even when the mortality cost of dispersal is the same for individuals of each sex (figure 1*b*).

Inclusive fitness is the sum of an individual's direct fitness (accrued through their impact on their own fitness) and indirect fitness (accrued through their impact on the fitness of their genetic relatives; [42]). A subadult female increases her inclusive fitness by dispersing away from her natal group when:3.1
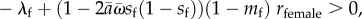
where 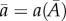 is the population average probability of a group initiating war, 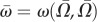 is the population average probability of the group winning the war, and *r*_female_ is the subadult female's relatedness to other females born in her natal group. That is, she suffers a direct-fitness cost (first term in condition (3.1)), owing to the probability *λ*_f_ of dying on the way to her new group. And she receives an indirect-fitness benefit (second term), owing to the relaxation of competition for breeding positions among females, to whom she may be genetically related, in her natal group. Specifically: with probability 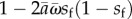 the female who wins the breeding position that she might otherwise have taken derives from her natal group, post-dispersal, as opposed to an attacking group; with probability 1 − *m*_f_ a female in her natal group, post-dispersal, was born in that same group, as opposed to migrating from elsewhere; and the relatedness between two females born in the same group is *r*_female_. Note that the fitness effects in condition (3.1) are correct up to a scaling factor that cancels out of the expression. An intermediate, convergence-stable [43,54] level of female dispersal *d*_f_* obtains when the left-hand side (l.h.s.) of condition (3.1) equals zero.

Analogously, a subadult male increases his inclusive fitness by dispersing away from his natal group when:3.2
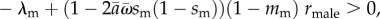
where *r*_male_ is his relatedness to the other males born in his natal group. An intermediate, convergence-stable level of male dispersal 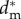 obtains when the l.h.s. of condition (3.2) equals zero.

Inspection of conditions (3.1) and (3.2) reveals that sex-biased dispersal may be favoured in two different ways. Firstly, the direct-fitness cost of dispersal may differ for the two sexes (*λ*_f_ ≠ *λ*_m_) such that, all else being equal, dispersal is more favoured in the sex with the lower cost (i.e. 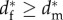
if *λ*_f_ ≤ *λ*_m_ and 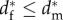
if *λ*_f_ ≥ *λ*_m_; figure 1*a*). This sex bias in dispersal translates into a sex bias in migration, in the same direction (figure 1*a*). Secondly, even if the direct-fitness cost is the same for both sexes (i.e. *λ*_f_ = *λ*_m_), the indirect-fitness benefit of dispersal may differ for the two sexes, owing to sex differences in the ecology of warfare. Specifically, denoting the extent to which the mothers of offspring born in a conquered group are a mixture of individuals from that group and from the conquering group (maternal admixture) by 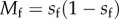, and the extent to which the fathers of offspring born in a conquered group are a mixture of individuals from that group and from the conquering group (paternal admixture) by 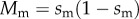, then—all else being equal—dispersal is more favoured in the sex with the lowest degree of admixture (i.e. 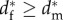 if *M*_f_ < *M*_m_ and 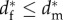 if *M*_f_ > *M*_m_; figure 1*b*). This sex bias in dispersal translates into a sex bias in migration, in the same direction (figure 1*b*). For example, if half of all offspring born into conquered groups are fathered by males of the conquered group and the other half are fathered by males from the conquering group (i.e. *s*_m_ = 0.5), but the mothers of all of these offspring are from the conquered group (i.e. *s*_f_ = 1.0), then there is less maternal admixture (i.e. *M*_f_ = 0.0) than there is paternal admixture (i.e. *M*_m_ = 0.25) and, consequently, females are relatively more favoured to disperse than are males (i.e. 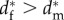). In this instance, a dispersing female is relatively more likely (and a dispersing male relatively less likely) to free up a breeding position for a relative, such that females obtain greater indirect-fitness benefits from dispersing. We confirm the robustness of these analytical results using individual-based simulations (see the electronic supplementary material for details).

### (b) Belligerence and bravery

The propensity of a group to go to war is determined by the average belligerence of its subadult males; and success in war is linked to these males' average bravery. However, although these two traits are expressed in subadult males, they may be controlled by various parties—including the male himself, his mother, his father, his maternal-origin genes, and his paternal-origin genes—whose inclusive-fitness interests may disagree with each other (intrafamily and intragenomic conflict). To assess the interests of these various parties, we hypothetically grant full control to each of them, in turn, and assess when an increase in the male's trait leads to an increase in the controller's inclusive fitness. We first describe the inclusive-fitness consequences of these traits under the influence of a general controller, before focusing on each control option in turn.

Participation in warfare implies fitness costs for the subadult male, but may result in fitness benefits for his subadult groupmates. Accordingly, the controller of the male's behaviour may derive an overall inclusive-fitness benefit by increasing his participation in warfare, depending upon how closely related the controller is to the male and how closely related the controller is to the male's groupmates. Specifically, the controller increases their inclusive fitness by increasing the male's level of belligerence when:3.3
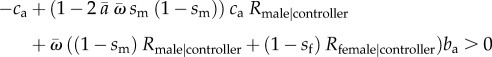
where *R*_male|controller_ is the relatedness of the controller to a random male groupmate of the focal male, expressed relative to the controller's relatedness to the focal male himself, and *R*_female|controller_ is the relatedness of the controller to a random female groupmate, expressed relative to the controller's relatedness to the focal male himself. That is, an increase in the focal male's belligerence leads: to an inclusive-fitness cost (first term in condition (3.3)), owing to the focal male's loss of competitiveness −*c*_a_ for breeding positions; an inclusive-fitness benefit (second term), owing to the *c*_a_ vacated breeding positions being occupied by other males, who are derived from the same group with probability 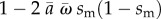 and, in which case, are related to the controller by *R*_male|controller_; an inclusive-fitness benefit (third term), owing to the increased probability *b*_a_ of going to war, which is won with probability 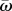 and consequently yields an extra 1 − *s*_m_ breeding success for male groupmates who are related to the controller by *R*_male|controller_ and an extra 1 − *s*_f_ breeding success for female groupmates who are related to the controller by *R*_female|controller_. Again, the fitness effects in condition (3.3) are correct up to a scaling factor that cancels out. Providing it takes an intermediate value, the convergence-stable level of belligerence is obtained by setting the l.h.s. of condition (3.3) equal to zero and solving for *Ā*=A*_controller_, which may be interpreted as the controller's belligerence optimum.

Similarly, the controller increases their inclusive fitness by increasing the male's level of bravery when:3.4
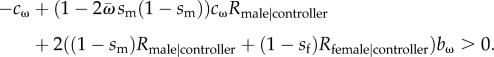


That is, an increase in the focal male's bravery leads: to an inclusive-fitness cost (first term in condition (3.4)), owing to the focal male's loss of competitiveness 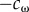 for breeding positions; an inclusive-fitness benefit (second term), owing to the 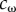 vacated breeding positions being occupied by other males, who are derived from the same group with probability 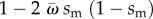 and, in which case, are related to the controller by *R*_male|controller_; an inclusive-fitness benefit (third term), owing to the increased probability *b_ω_* of winning a war and consequently yielding an extra 1 − *s*_m_ breeding success for male groupmates who are related to the controller by *R*_male|controller_, and an extra 1 − *s*_f_ breeding success for female groupmates who are related to the controller by *R*_female|controller_. Once again, the fitness effects in condition (3.4) are correct up to a scaling factor. Providing it takes an intermediate value, the convergence-stable level of bravery is obtained by setting the l.h.s. of condition (3.4) equal to zero and solving for 
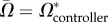
, which may be interpreted as the controller's bravery optimum.

### (c) Intrafamily conflict

**Figure 2.**
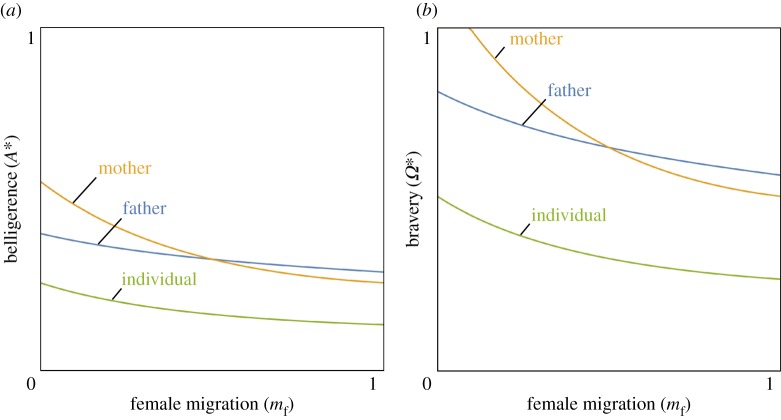
Intrafamily conflicts over belligerence and bravery. Convergence-stable levels of belligerence (A*, (*a*)) and bravery (Ω*, (*b*)) as functions of female migration (*m*_f_) when belligerence is controlled by the focal male's father (blue line), his mother (orange line), or the focal male himself (green line). Other parameter values are 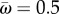 ((*a*) only), 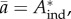 ((*b*) only), *m*_m_ = 0.5, *s*_f_ = 1, *s*_m_ = 0, *N*_f_ = *N*_m_ = 10. We assume functional forms *a* = *A*_att_ and *t* = 1–0.025 *a*^2^ (*a*), and *ω*(*Ω*_att_, *Ω*_def_) = ½ (1 + *Ω*_att_ – *Ω*_def_) and *τ* = 1 – 0.025 *Ω*^2^ (*b*). (Online version in colour.)

Different members of the family may come into conflict over social behaviour [26,55–57] and in ways that are modulated by patterns of dispersal (e.g. [58]). Here, we consider the inclusive-fitness interests of the subadult male, his mother, and his father, in relation to the optimal levels of belligerence and bravery that he should express. For ease of presentation, for the remainder of our analysis we focus upon a scenario in which there are equal numbers of male and female breeders in each group (*N*_f_ = *N*_m_ = *N*) and all offspring born in conquered groups are begot by mothers from the conquered group (*s*_f_ = 1) and by males from the conquering group (*s*_m_ = 0), and we treat migration rates as fixed parameters (as in [7]; see the electronic supplementary material for more general results and demonstration that all combinations of sex-specific migration rates are evolutionarily feasible). We find that parents always favour a higher level of altruism in warfare than do their sons and that mothers and fathers disagree when there is a sex-bias in migration (figure 2).

The conditions (3.3) and (3.4) under which increases in belligerence and bravery are favoured depend on relatedness coefficients *R*_male**|**controller_ and *R*_female**|**controller_, which may be different for different controllers. Accordingly, different controllers may have different belligerence (A*) and bravery (Ω*) optima. If a male's behaviour is controlled by his father, these relatedness coefficients above are given by *R*_male|father_ and *R*_female|father_, which may be expressed in terms of model parameters (table 1). Substituting these relatedness coefficients into conditions (3.3) and (3.4), we can determine belligerence 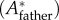 and bravery 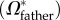 optima from the perspective of the subadult male's father, and this recovers the results reported by Lehmann & Feldman [7,59] (see the electronic supplementary material for details; figure 2). Alternatively, if the male's behaviour is controlled by his mother, relatedness is given by *R*_male|mother_ and *R*_female|mother_ (table 1). Substituting these relatedness coefficients into conditions (3.3) and (3.4), we can determine belligerence 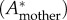 and bravery 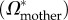 optima from the perspective of the subadult male's mother (figure 2). Finally, if the male's behaviour is under his own, individual control, relatedness is given by *R*_male|individual_ and *R*_female|individual_ (table 1). Substituting these relatedness coefficients into conditions (3.3) and (3.4), we can determine belligerence 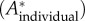 and bravery 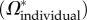 optima from the perspective of the subadult male himself (figure 2).

**Table 1.** Relatedness. Coefficients of relatedness *R*_recipient*|*controller_ between the controller of a male's behaviour (the individual male himself, his father, his mother, his genes of unknown parental origin, his paternal-origin genes, and his maternal-origin genes) and the recipients whose fitnesses are modulated by this behaviour (male groupmates and female groupmates) in the context of belligerence and bravery behaviours. These coefficients depend upon the relatedness of two subadults born in the same group, i.e. 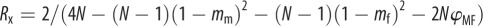, and the probability that two adults of opposite sex in the same post-competition group were born in the same group, i.e. 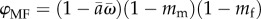.

controller	recipient	
	male	female
individual	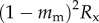	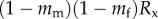
father	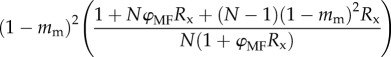	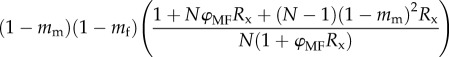
mother	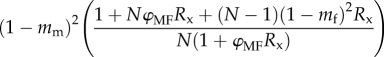	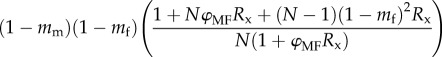
unknown	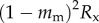	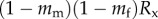
paternal	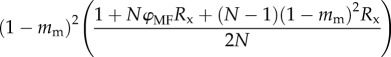	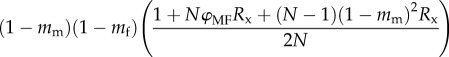
maternal	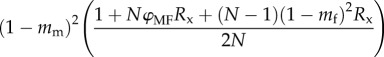	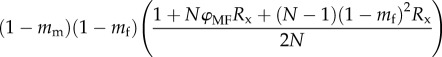

Comparison of these relatedness coefficients reveals two key results. Firstly, a subadult male is always less related to other subadults in his group than are his parents (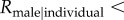 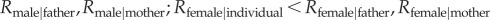), such that his belligerence and bravery optima are always lower than those of his parents (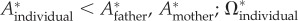

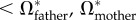
; figure 2). Secondly, the relatedness coefficients from his father's and mother's perspectives coincide only when migration is unbiased (*m*_f_ = *m*_m_); when migration is female-biased (*m*_f_ > *m*_m_), his mother is less related than his father to his groupmates (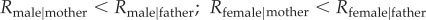) and, consequently, his mother favours less belligerence and bravery than does his father (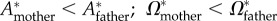); and when migration is male-biased (*m*_f_ < *m*_m_), his mother is more related than his father to his groupmates (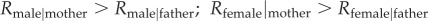) and, consequently, his mother favours more belligerence and bravery than does his father (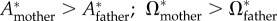; figure 2). We confirm the robustness of these analytical results using individual-based simulations (see the electronic supplementary material for details).

### (d) Intragenomic conflict

**Figure 3.**
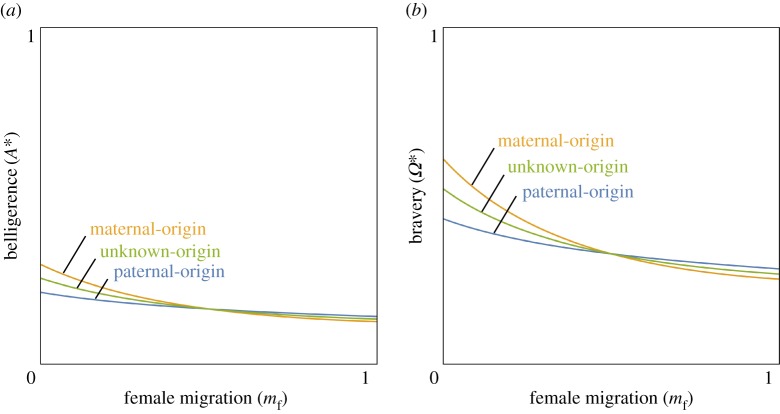
Intragenomic conflicts over belligerence and bravery. Convergence-stable level of belligerence (A*, (*a*)) and bravery (Ω*, (*b*)) as functions of female migration (*m*_f_) when belligerence or bravery are controlled by the focal individual's paternal-origin genes (blue line), maternal-origin genes (orange line), or unknown-origin genes (green line). Other parameter values are 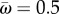 ((*a*) only), 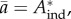 ((*b*) only) and *m*_m_ = 0.5, *s*_f_ = 1, *s*_m_ = 0, *N*_f_ = *N*_m_ = 10. We assume functional forms *a* = *A*_att_ and *t* = 1–0.025 *a*^2^ (*a*), and *ω*(*Ω*_att_, *Ω*_def_) = ½ (1 + *Ω*_att_ − *Ω*_def_) and *τ* = 1–0.025 *Ω*^2^ (*b*). (Online version in colour.)

Sex-biased demography has been implicated in intragenomic conflicts for a variety of social behaviours [28–33,60–62]. Here, we investigate the potential for conflict over belligerence and bravery within the male's genome, by considering the inclusive-fitness interests [42,63,64] of his maternal-origin genes, paternal-origin genes, and genes of unknown parental origin (figure 3). If the male's behaviour were fully controlled by his paternal-origin genes, relatedness coefficients *R*_male**|**controller_ and *R*_female**|**controller_ in conditions (3.3) and (3.4) would be given by *R*_male|paternal_ and *R*_female|paternal_ (table 1). Substituting these relatedness coefficients into conditions (3.3) and (3.4), we can determine belligerence 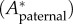 and bravery 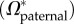 optima from the perspective of the subadult male's paternal-origin genes (figure 3). Alternatively, if the male's behaviour were fully controlled by his maternal-origin genes, relatedness would be given by *R*_male|maternal_ and *R*_female|maternal_ (table 1). Substituting these relatedness coefficients into conditions (3.3) and (3.4), we can determine the belligerence 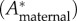 and bravery 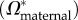 optima from the perspective of the subadult male's maternal-origin genes (figure 3). Finally, relatedness for a gene of unknown parental origin is given by *R*_male|unknown_ and *R*_female|unknown_ (table 1). Note that these exactly coincide with the relatedness coefficients for the individual carrying the genes, *R*_male|individual_ and *R*_female|individual_ (table 1). Substituting these relatedness coefficients into conditions (3.3) and (3.4), we can determine belligerence 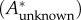 and bravery 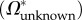 optima from the perspective of the subadult male's genes of unknown parental origin (figure 3).

Comparison of relatedness coefficients yields two further key results. Firstly, relatedness for a gene of unknown parental origin is the arithmetic mean of those for maternal- and paternal-origin genes [36] and, accordingly, the belligerence or bravery optimum for a gene of unknown parental origin is always intermediate between those of maternal- and paternal-origin genes (figure 3). Secondly: the relatedness coefficients for a male's maternal-origin and paternal-origin genes coincide only when migration is unbiased (*m*_f_ = *m*_m_); when migration is female-biased (*m*_f_ > *m*_m_), his maternal-origin genes are less related than his paternal-origin genes to his groupmates (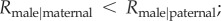 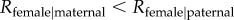) and, consequently, his maternal-origin genes favour less belligerence and bravery than do his paternal-origin genes (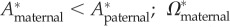

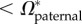
); and when migration is male-biased (*m*_f_ < *m*_m_), his maternal-origin genes are more related than his paternal-origin genes to his groupmates (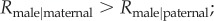 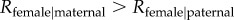) and, consequently, his maternal-origin genes favour more belligerence and bravery than do his paternal-origin genes (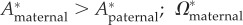 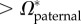; figure 3). We confirm the robustness of these analytical results using individual-based simulations (see the electronic supplementary material for details).

### (e) Genomic imprinting

The kinship theory of genomic imprinting suggests that intragenomic conflicts between maternal-origin and paternal-origin genes drive the evolution of parent-of-origin-specific gene expression [36,37,65]. According to the ‘loudest voice prevails' principle [36], this conflict ultimately leads to self-imposed silencing of one of the genes. Specifically, if the locus of interest encodes a gene product that promotes the contested phenotype, then the gene with the higher phenotypic optimum is favoured to upregulate its level of expression, while the gene with the lower optimum is favoured to downregulate its expression, and this antagonistic escalation results in the latter gene silencing itself and the former gene expressing at its desired level. By contrast, if the locus encodes a gene product that inhibits the contested phenotype, then it is the gene with the higher phenotypic optimum that is predicted to silence itself and the other gene to express at its desired level.

**Figure 4.**
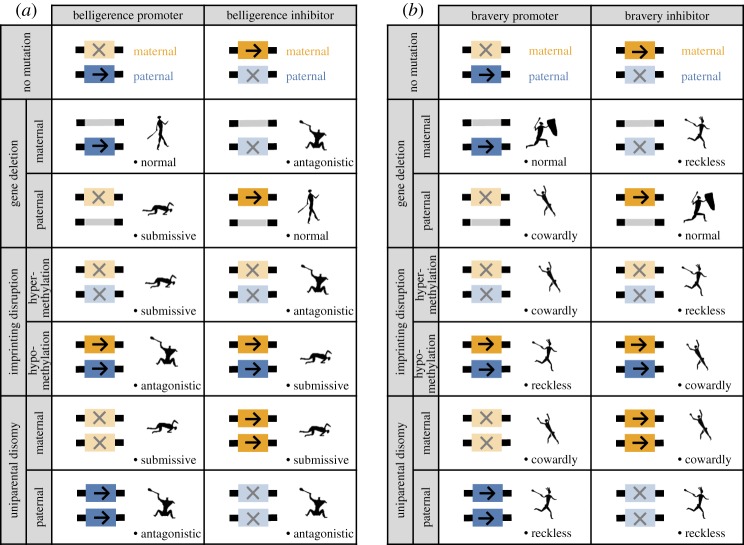
Genomic imprinting and associated pathologies. Predicted patterns of parent-of-origin-specific gene expression and concomitant phenotypes for loci that are either promoters or inhibitors of belligerence (*a*) or bravery (*b*), under normal conditions and also as a result of three different mutational or epimutational perturbations: gene deletion, imprinting disruption, or uniparental disomy. Genes are either of maternal-origin (orange) or paternal-origin (blue) and are either silenced (crosses) or expressed (arrows). Human figures from the George Stow collection at Iziko South African Museum, derived from *The Digital Bleek and Lloyd* (http://lloydbleekcollection.cs.uct.ac.za/) with permission. (Online version in colour.)

The loudest voice prevails principle may be used to make predictions as to patterns of gene expression for loci underlying belligerence and bravery phenotypes (figure 4). For simplicity, we focus on the case in which relatedness is higher for paternal-origin genes than for maternal-origin genes (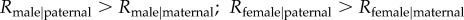), e.g. owing to female-biased dispersal. In this scenario, paternal-origin genes favour more belligerence and bravery than do maternal-origin genes (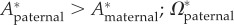

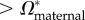
). Exactly the opposite patterns are obtained if relatedness is higher for maternal-origin genes than for paternal-origin genes. Considering a locus for which the gene product acts to increase belligerence (i.e. a ‘belligerence promoter’), as the maternal-origin gene favours less belligerence than does the paternal-origin gene, we predict the former to be silenced and the latter to be expressed (figure 4*a*). By contrast, considering a locus for which the gene product acts to decrease belligerence (i.e. a ‘belligerence inhibitor’), we predict the paternal-origin gene to be silenced and the maternal-origin gene to be expressed (figure 4*a*). Analogously, we predict that a bravery promoter will be maternally silenced and paternally expressed (figure 4*b*) and that a belligerence inhibitor will be paternally silenced and maternally expressed (figure 4*b*).

### (f) Associated pathologies

Genomic imprinting results in functional haploidy, rendering the individual vulnerable to a range of deleterious mutations and epimutations [38]. These might have no visible effect or, alternatively, lead to abnormal phenotypes and pathological conditions that are very far from realizing the inclusive-fitness interests of either maternal-origin or paternal-origin genes [29]. Here, we consider three different types of perturbations: (i) a gene deletion (or, equivalently, a point mutation resulting in a non-functional gene product, or an experimentally induced knockout), (ii) a malfunctioning of the imprinting machinery, whereby the addition of methyl tags to genes that are normally expressed leads to erroneous silencing (hyper-methylation), or the absence of methyl tags from genes that are normally silenced leads to erroneous expression (hypo-methylation; [35]), and (iii) uniparental disomy, whereby both of the individual's genes derive from one parent. Again, for compactness of presentation, we only consider female-biased dispersal.

Considering a belligerence promoter, which is expected to be maternally silenced and paternally expressed: deletion of the maternal-origin gene has no effect and leads to a normal phenotype; deletion of the paternal-origin gene results in the complete absence of gene product and hence an abnormally low level of belligerence (‘submissive’ phenotype); hyper-methylation silences the paternal-origin gene, resulting in the submissive phenotype; hypo-methylation activates the maternal-origin gene, resulting in an ‘antagonistic’ phenotype; maternal disomy results in the complete absence of gene product, and hence the submissive phenotype; and paternal disomy results in an abnormally large amount of gene product, and hence the antagonistic phenotype (figure 4*a*). By contrast, considering a belligerence inhibitor, which is expected to be paternally silenced and maternally expressed: deletion of the maternal-origin gene results in the complete absence of gene product and hence the antagonistic phenotype; deletion of the paternal-origin gene results in the normal phenotype; hyper-methylation silences the maternal-origin gene, resulting in the antagonistic phenotype; while hypo-methylation activates the paternal-origin gene, resulting in the submissive phenotype; maternal disomy results in an abnormally high amount of gene product, and hence the submissive phenotype; and paternal disomy results in the complete absence of the gene product, and hence the antagonistic phenotype (figure 4*a*). Exactly analogous patterns obtain for bravery genes, with mutations and epimutations variously giving rise to abnormally low levels of bravery (‘cowardly’ phenotype), abnormally high levels of bravery (‘reckless’ phenotype), or a normal phenotype (figure 4*b*).

## 4. Discussion

Despite huge interest in the evolution of warfare, conflicts both between family members and within the warring individual have been relatively neglected. Here, we developed and analysed a model of warfare in the context of sex-biased demography. We found that the ecology of war can drive the evolution of sex-biased dispersal. Moreover, we found that these same patterns of sex-biased dispersal can modulate intrafamily and intragenomic conflicts over warfare, and accordingly parent-of-origin-specific patterns of gene expression—i.e. ‘genomic imprinting’—and concomitant mutational and epimutational pathologies in relation to intergroup violence phenotypes.

We have shown that sex-biased dispersal can be driven by sexual asymmetries in the spoils of war. In particular, insofar as warfare enables males extra opportunities to compete with non-kin for breeding positions—thus relaxing kin competition—to an extent that is greater than for females, then males are less strongly favoured to disperse as a means of reducing kin competition for breeding positions (cf. [66,67]) and this may result in the evolution of female-biased dispersal (patrilocality). This ecology-of-war effect provides a novel potential explanation for the unusual female-biased dispersal of ancestral humans [49], modern hunter–gatherers [51], and African apes, especially chimpanzees [50], which contrasts with the male-biased dispersal observed in most mammals [68,69]. In addition, we have shown that female-biased dispersal may be favoured when females suffer a lower cost of dispersal (cf. [70]), e.g. owing to a greater likelihood that they will be accepted into a new group, as has been reported in primates [50]. Considered together, these two results suggest that male–male violence—in the context of war and/or against immigrants—may have been a key driver of patrilocality.

Our analysis suggests that intense intrafamily conflict may arise in the context of warfare, with parents encouraging reluctant sons to go to war and to show valour in battle (on account of the inclusive-fitness costs of warfare being lower for parents than for sons, it being the latter whose lives are at risk), and with fathers being more encouraging and mothers being more circumspect (on account of the inclusive-fitness benefits of warfare being higher for fathers than for mothers, the former being—on average—more related to the local group). Such conflicts of interests underline the importance of understanding the cultural transmission of warfare (reviewed in [71]): in particular, boys learning about war from their fathers—e.g. as occurs in the Jivaro of South America and the Mae Enga of Papua New Guinea [71] among others—suggests a means by which fathers may exert control over their sons' conduct in relation to warfare. More generally, influence may extend beyond the family, such as when leaders decide the behaviour of their followers [72]. These points highlight that genetics and culture need not always provide competing explanations for warfare (*contra* [71,73]), but rather cultural transmission and social norms may provide avenues for different genetic parties to exert their influence over human behaviour.

We have also shown that sex-specific demography can generate intragenomic conflict over warfare. Specifically, female-biased dispersal can result in a young male being more related to his groupmates via his father than via his mother, such that his paternal-origin genes are relatively more favoured to induce belligerence and bravery behaviours than are his maternal-origin genes. We predict that this intragenomic conflict will result in genomic imprinting: loci that promote belligerence and/or bravery behaviours are expected to be maternally silenced and paternally expressed, while loci that inhibit these behaviours are expected to be paternally silenced and maternally expressed (figure 4). Although our main focus has been on female-biased dispersal, other sex-specific demographies are expected to yield similar predictions (cf. [29–32]). These include higher male variance in reproductive success (e.g. owing to polygyny; cf. [74]) and higher male mortality (e.g. owing to male–male violence; [24]). Importantly, our predictions are expected to be relatively robust to quantitative variation in these sex-specific parameters, as the existence and direction of imprint depends only on the existence and direction—and not the magnitude—of intragenomic conflict [33].

A remarkable feature of the kinship theory of genomic imprinting is that it not only illuminates adaptation but also yields testable predictions as to the particular maladaptive phenotypes associated with deleterious genetic and epigenetic mutations [29,30,32,62,75–77]. We have shown that mutations and epimutations tilting the balance towards paternally expressed belligerence and bravery loci are expected to result in ‘aggressive’ and ‘reckless’ pathologies, while those tilting the balance towards maternally expressed loci are expected to result in ‘submissive’ and ‘cowardly’ pathologies, these being extreme phenotypes that lie far beyond the inclusive-fitness optima of any of the individual's genes. Accordingly, our analysis suggests that some instances of societally damaging intergroup violence may represent maladaptive defects rather than well-honed adaptations to our ancestral environment. Understanding that such violence may be associated with imprinting disorders should facilitate discovery of the genes involved.

## Supplementary Material

Supporting Information

## Supplementary Material

Simulation codes
