# Supplementary material for: Intrafamily and intragenomic conflicts in human warfare
Source: Proc Biol Sci. 2017 Feb 22;284(1849):20162699. doi: 10.1098/rspb.2016.2699 (PMC5326533; doi:10.1098/rspb.2016.2699)
Supplement: Supporting Information [file rspb20162699supp1.pdf]

Supporting Information for

# Intrafamily and intragenomic conflicts in human warfare

Alberto J. C. Micheletti, Graeme D. Ruxton & Andy Gardner

## Contents

**1 Fitness** .......... **2**

**2 Female dispersal** .......... **4**

2.1 Marginal fitness .......... 4

2.2 Consanguinity and relatedness .......... 5

**3 Male dispersal** .......... **6**

3.1 Marginal fitness .......... 6

**4 Sex-biased dispersal and migration** .......... **7**

4.1 Conditions for sex-biased dispersal .......... 7

4.2 Convergence stability of migration rates .......... 10

4.3 Individual-based simulations for dispersal evolution .......... 11

**5 Belligerence** .......... **16**

5.1 Marginal fitness .......... 16

5.2 Consanguinity and relatedness .......... 18

5.3 Individual-based simulations for belligerence evolution .......... 20

**6 Bravery** .......... **23**

6.1 Marginal fitness .......... 23

6.2 Individual-based simulations for bravery evolution .......... 24

**7 Additional references** .......... **27**

**8 Tables** .......... **28**

## 1 Fitness

We begin by deriving the fitness of a focal subadult male in a focal group. We consider fitness under all possible dispersal decisions, events and outcomes of war, following the life cycle described in Methods and represented in Figure A1.1. With probability $1 - d_m$, the focal male does not disperse and remains in the focal group. In every generation, each post-dispersal group is in a position to attack one randomly-chosen group and to be attacked by one other group. The focal group is not attacked by the other group with probability $1 - \bar{a}$, in which case the focal male competes for $N_m$ breeding spots with $N_f K_m((1 - d'_m) + \bar{d}_m (1 - \lambda_m))\, t(A')$ males, where $t(A')$ is the group-average competitiveness (with $dt/dA < 0$, and $t(0) = 1$). Considering that the competitiveness of the focal male due to belligerence is $t(A)$, his probability of securing a breeding spot in this case is $(N_m\, t(A))/(N_f K_m ((1 - d'_m) + \bar{d}_m (1 - \lambda_m))\, t(A'))$. Alternatively, the focal group is attacked with probability $\bar{a}$ and the attacking group loses the war with probability $1 - \omega''$, where $\omega'' \equiv \omega(\bar{\Omega}, \Omega')$. In this case, the probability of survival of the average male in the group is multiplied by $\tau(\Omega')$ and that of the focal male by $\tau(\Omega)$, where $\tau(\Omega_{ind})$ is competitiveness due to bravery (with $d\tau/d\,\Omega < 0$, and $\tau(0) = 1$). Therefore, in this case, the focal male secures a breeding spot with probability $(N_m\, t(A)\, \tau(\Omega))/(N_f K_m ((1 - d'_m) + \bar{d}_m (1 - \lambda_m))\; t(A')\, \tau(\Omega'))$. We assume that the competitiveness terms due to belligerence and bravery are multiplicative for mathematical convenience. Otherwise, the attacking group wins the war with probability $\omega''$ and in this case the focal male competes for $N_m\, s_m$ breeding spots with $N_f K_m ((1 - d'_m) + \bar{d}_m (1 - \lambda_m))\, s_m$ males from his group and $N_f K_m ((1 - \bar{d}_m) + \bar{d}_m (1 - \lambda_m))\, (1 - s_m)$ males from the attacking group. Adding the appropriate competitiveness modifiers, the overall probability that the focal male secures a spot in this case is $(N_m\, t(A)\, \tau(\Omega)\, s_m)$ / $(N_f K_m((1 - d'_m) + \bar{d}_m (1 - \lambda_m))\, t(A')\, \tau(\Omega')\, s_m + N_f K_m ((1 - \bar{d}_m) + \bar{d}_m (1 - \lambda_m))\, t(\bar{A})\, \tau(\bar{\Omega}))\, (1 - s_m))$. In addition, the focal group has the opportunity to attack one other group. With probability $1 - a'$, where $a' = a(A')$, this does not happen, and in this case the focal male does not get access to additional breeding spots. Alternatively, the focal group goes to war with probability $a'$ and loses with probability $1 - \omega'$, where $\omega' = \omega(\Omega', \bar{\Omega})$, which again results in no additional breeding spots for the focal male. Otherwise, the focal group wins with probability $\omega'$ in which case the focal male has access to an additional $N_m (1 - s_m)$ breeding spots, for which he competes with $N_f K_m ((1 - d'_m) + \bar{d}_m (1 - \lambda_m))(1 - s_m)$ males from his group and $N_f K_m((1 - \bar{d}_m) + \bar{d}_m (1 - \lambda_m))\, s_m$ males from the conquered group. Taking into account the competitiveness due to belligerence and bravery as before, the probability that the focal male secures a breeding spot in this case is equal to $(N_m\; t(A)\, \tau(\Omega)\, (1 - s_m))$ / $(N_f K_m ((1 - d'_m) + \bar{d}_m (1 - \lambda_m))\, t(A')\, \tau(\Omega')\, (1 - s_m) + N_f K_m ((1 - \bar{d}_m) + \bar{d}_m (1 - \lambda_m))\, t(\bar{A})\, \tau(\bar{\Omega})\, s_m)$. Alternatively, the focal male disperses from the natal group with probability $d_m$ and successfully reaches and becomes part of another group with probability $1 - \lambda_m$. In this case, fitness is derived analogously to the philopatry case, with the exception that the number of males in the focal group is equal to $N_f K_m ((1 - \bar{d}_m) + \bar{d}_m (1 - \lambda_m))$. Therefore, the absolute fitness of a focal subadult male is equal to:

$$w_m = \Bigg( (1 - d_m) \Bigg( (1 - \bar{a}) \frac{t(A)}{\big((1 - d'_m) + \bar{d}_m (1 - \lambda_m)\big) t(A')} + \bar{a} \Bigg( (1 - \omega'') \frac{t(A)\,\tau(\Omega)}{\big((1 - d'_m) + \bar{d}_m (1 - \lambda_m)\big) t(A') \tau(\Omega')} +$$

$$\omega'' \frac{t(A) \tau(\Omega) s_m}{\big((1 - d'_m) + \bar{d}_m (1 - \lambda_m)\big) t(A') \tau(\Omega') s_m + \big((1 - \bar{d}_m) + \bar{d}_m (1 - \lambda_m)\big) t(\bar{A})\; \tau(\bar{\Omega}) (1 - s_m)} \Bigg) +$$

$$a'\,\omega'\frac{t(A)\,\tau(\Omega)(1-s_{\mathrm{m}})}{\big((1-d'_{\mathrm{m}})+\bar{d}_{\mathrm{m}}(1-\lambda_{\mathrm{m}})\big)t(A')\tau(\Omega')(1-s_{\mathrm{m}})+\big((1-\bar{d}_{\mathrm{m}})+\bar{d}_{\mathrm{m}}(1-\lambda_{\mathrm{m}})\big)t(\bar{A})\,\tau(\bar{\Omega})s_{\mathrm{m}}}\Bigg)+d_{\mathrm{m}}(1-\lambda_{\mathrm{m}})\Bigg((1-$$

$$\bar{a})\frac{t(A)}{t(A')}\frac{1}{(1-\bar{d}_{\mathrm{m}})+\bar{d}_{\mathrm{m}}(1-\lambda_{\mathrm{m}})}+\bar{a}\Bigg((1-\omega'')\frac{t(A)\,\tau(\Omega)}{t(A')\tau(\Omega')}\frac{1}{(1-\bar{d}_{\mathrm{m}})+\bar{d}_{\mathrm{m}}(1-\lambda_{\mathrm{m}})}+$$

$$\omega''\frac{t(A)\,\tau(\Omega)s_{\mathrm{m}}}{\big((1-\bar{d}_{\mathrm{m}})+\bar{d}_{\mathrm{m}}(1-\lambda_{\mathrm{m}})\big)t(A')\tau(\Omega')s_{\mathrm{m}}+\big((1-\bar{d}_{\mathrm{m}})+\bar{d}_{\mathrm{m}}(1-\lambda_{\mathrm{m}})\big)t(\bar{A})\,\tau(\bar{\Omega})(1-s_{\mathrm{m}})}\Bigg)+$$

$$\bar{a}\bar{\omega}\frac{t(A)\,\tau(\Omega)(1-s_{\mathrm{m}})}{\big((1-\bar{d}_{\mathrm{m}})+\bar{d}_{\mathrm{m}}(1-\lambda_{\mathrm{m}})\big)t(A')\tau(\Omega')(1-s_{\mathrm{m}})+\big((1-\bar{d}_{\mathrm{m}})+\bar{d}_{\mathrm{m}}(1-\lambda_{\mathrm{m}})\big)\,t(\bar{A})\,\tau(\bar{\Omega})s_{\mathrm{m}}}\Bigg)\Bigg)\frac{N_{\mathrm{m}}}{N_{\mathrm{f}}K_{\mathrm{m}}}. \qquad \text{(A1)}$$

The average fitness of subadult males in the population is $\bar{w}_{\mathrm{m}} = N_{\mathrm{m}}/(K_{\mathrm{m}}\,N_{\mathrm{f}})$. Therefore, the relative fitness of the focal subadult male is given by $W_{\mathrm{m}} = w_{\mathrm{m}} / \bar{w}_{\mathrm{m}}$. Similarly, the absolute fitness of a focal subadult female can be written as:

$$w_{\mathrm{f}}=\Bigg((1-d_{\mathrm{f}})\Bigg((1-\bar{a})\frac{1}{(1-d'_{\mathrm{f}})+\bar{d}_{\mathrm{f}}(1-\lambda_{\mathrm{f}})}+\bar{a}\Bigg((1-\omega'')\frac{1}{(1-d'_{\mathrm{f}})+\bar{d}_{\mathrm{f}}(1-\lambda_{\mathrm{f}})}+$$

$$\omega''\frac{s_{\mathrm{f}}}{\big((1-d'_{\mathrm{f}})+\bar{d}_{\mathrm{f}}(1-\lambda_{\mathrm{f}})\big)s_{\mathrm{f}}+\big((1-\bar{d}_{\mathrm{f}})+\bar{d}_{\mathrm{f}}(1-\lambda_{\mathrm{f}})\big)(1-s_{\mathrm{f}})}\Bigg)+a'\,\omega'\frac{(1-s_{\mathrm{f}})}{\big((1-d'_{\mathrm{f}})+\bar{d}_{\mathrm{f}}(1-\lambda_{\mathrm{f}})\big)(1-s_{\mathrm{f}})+\big((1-\bar{d}_{\mathrm{f}})+\bar{d}_{\mathrm{f}}(1-\lambda_{\mathrm{f}})\big)s_{\mathrm{f}}}\Bigg)+d_{\mathrm{f}}(1-$$

$$\lambda_{\mathrm{f}})\Bigg((1-\bar{a})\frac{1}{(1-\bar{d}_{\mathrm{f}})+\bar{d}_{\mathrm{f}}(1-\lambda_{\mathrm{f}})}+\bar{a}\Bigg((1-\omega'')\frac{1}{(1-\bar{d}_{\mathrm{f}})+\bar{d}_{\mathrm{f}}(1-\lambda_{\mathrm{f}})}+\omega''\frac{s_{\mathrm{f}}}{\big((1-\bar{d}_{\mathrm{f}})+\bar{d}_{\mathrm{f}}(1-\lambda_{\mathrm{f}})\big)s_{\mathrm{f}}+\big((1-\bar{d}_{\mathrm{f}})+\bar{d}_{\mathrm{f}}(1-\lambda_{\mathrm{f}})\big)(1-s_{\mathrm{f}})}\Bigg)+$$

$$\bar{a}\bar{\omega}\frac{(1-s_{\mathrm{f}})}{\big((1-\bar{d}_{\mathrm{f}})+\bar{d}_{\mathrm{f}}(1-\lambda_{\mathrm{f}})\big)(1-s_{\mathrm{f}})+\big((1-\bar{d}_{\mathrm{f}})+\bar{d}_{\mathrm{f}}(1-\lambda_{\mathrm{f}})\big)s_{\mathrm{f}}}\Bigg)\Bigg)\frac{1}{K_{\mathrm{f}}}\,. \qquad \text{(A2)}$$

Notice that females do not express the genes for belligerence and bravery and therefore do not pay the associated costs. The average fitness of subadult females in the population is $\bar{w}_{\mathrm{f}} = 1/K_{\mathrm{f}}$. Therefore, the relative fitness of the focal subadult female is given by $W_{\mathrm{f}} = w_{\mathrm{f}} / \bar{w}_{\mathrm{f}}$.

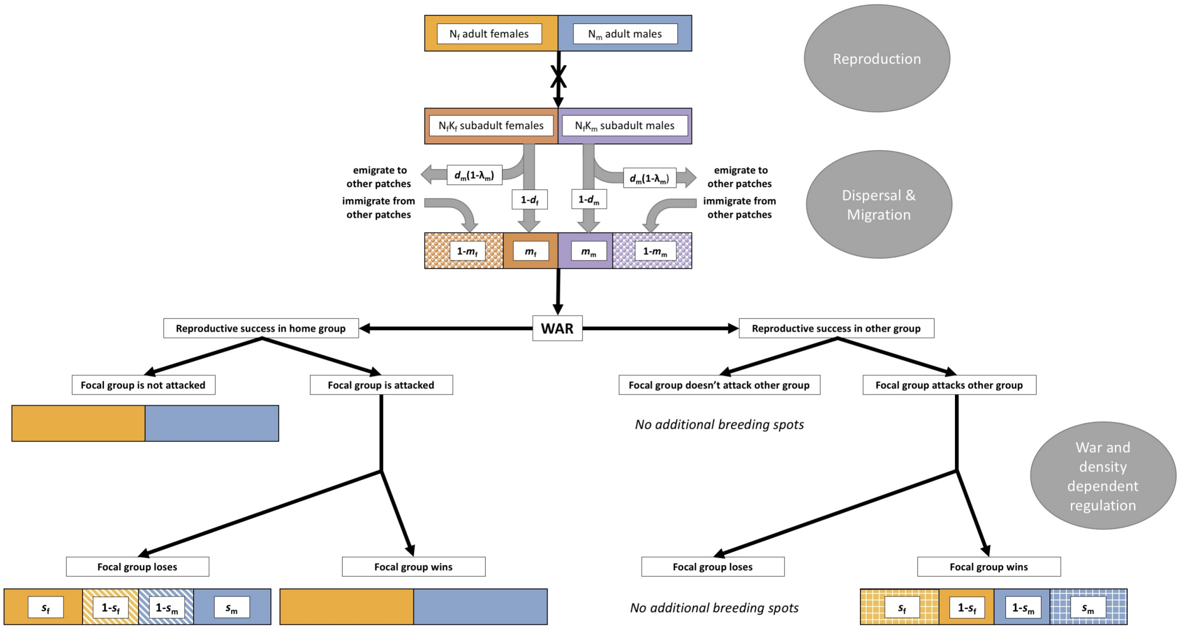


**Figure A1.1 – Life cycle as described in the Methods section. Orange and blue represent adult males and females, respectively; brown and purple represent subadult females and males, respectively.**

## 2 Female dispersal

### *2.1 Marginal fitness*

Consider a locus $\mathcal{G}$. We denote the genic value of the focal individual for this gene by $g$. Secondly, we denote the breeding value of the individual controlling the phenotype (the 'controller') by $G$, that of a groupmate's controller by $G'$, and the average of the population by $\bar{G}$. Note that, in the case in which the controller is the focal individual, $G$ is the breeding value of the focal individual. Consider that this locus controls female dispersal (the phenotype is exhibited by females, but not by males). Assuming vanishing genetic variation, meaning that all breeding values of the population are clustered around the mean [37,38], the direction of natural selection is given by:

$$\frac{\mathrm{d}W}{\mathrm{d}g} = c_\mathrm{f}\frac{\mathrm{d}W_\mathrm{f}}{\mathrm{d}g_\mathrm{f}} + c_\mathrm{m}\frac{\mathrm{d}W_\mathrm{m}}{\mathrm{d}g_\mathrm{m}}, \qquad \text{(A3)}$$

where: $W = c_\mathrm{f}\, W_\mathrm{f} + c_\mathrm{m}\, W_\mathrm{m}$ is an average of relative fitness, taken over female and male classes, with the class reproductive values $c_\mathrm{f}$ and $c_\mathrm{m}$ of females and males providing the appropriate weights [37,38,40,73,74]; and with all derivatives evaluated at $g = g_\mathrm{m} = g_\mathrm{f} = \bar{G}$.

The derivative $\mathrm{d}W_\mathrm{f}/\mathrm{d}g_\mathrm{f}$ describes how the genic value of a gene drawn from a subadult female impacts on her relative fitness. It depends on i) the association between the dispersal

phenotype of the focal female and her own relative fitness, the association between the breeding value for the dispersal gene of the female's controller and the focal female's dispersal phenotype, the association between the genic value of the focal female and the breeding value of her controller, and ii) the association between the dispersal phenotype of a random female in the focal group and the focal female's relative fitness, the association between the breeding value for the dispersal gene of the random female's controller and the random female's dispersal phenotype, the association between the genic value of the focal female and the breeding value of the random female's controller. This can be expressed mathematically as:

$$\frac{\mathrm{d}W_\mathrm{f}}{\mathrm{d}g_\mathrm{f}} = \frac{\partial W_\mathrm{f}}{\partial d_\mathrm{f}}\frac{\mathrm{d}d_\mathrm{f}}{\mathrm{d}G}\frac{\mathrm{d}G}{\mathrm{d}g_\mathrm{f}} + \frac{\partial W_\mathrm{f}}{\partial d_{\mathrm{f}'}}\frac{\mathrm{d}d_{\mathrm{f}'}}{\mathrm{d}G'}\frac{\mathrm{d}G'}{\mathrm{d}g_\mathrm{f}} = \left(\frac{\partial W_\mathrm{f}}{\partial d_\mathrm{f}}p_\mathrm{controller} + \frac{\partial W_\mathrm{f}}{\partial d_{\mathrm{f}'}}p_\mathrm{female}\right)\gamma\,, \tag{A4}$$

where $p_\mathrm{controller} = \mathrm{d}G/\mathrm{d}g_\mathrm{f}$ is the consanguinity between the focal female and her controller, $p_\mathrm{female} = \mathrm{d}G'/\mathrm{d}g_\mathrm{f}$ is the consanguinity between the controller of the focal female and a random female in the focal female's group, and $\gamma = \mathrm{d}d_\mathrm{f}/\mathrm{d}G = \mathrm{d}d_\mathrm{f}'/\mathrm{d}G'$ is the correlation between an individual's phenotype and its controller's breeding value.

The derivative $\mathrm{d}W_\mathrm{m}/\mathrm{d}g_\mathrm{m}$ describes how the genic value of a gene drawn from a subadult male impacts on his relative fitness. Since males carry the gene, but do not express it, and competition occurs within sexes, there is no impact on male fitness and this derivative is null.

The condition for an increase in population average female dispersal is $\mathrm{d}W/\mathrm{d}g > 0$ [37] and, as $c_\mathrm{f} = c_\mathrm{m} = 1/2$ under diploid inheritance [37,73,74], this condition is equivalent to:

$$-\frac{\lambda_\mathrm{f}}{1-\bar{d}_\mathrm{f}\,\lambda_\mathrm{f}}p_\mathrm{controller} + \frac{(1-\bar{d}_\mathrm{f})(1-2\bar{a}\bar{\omega}s_\mathrm{f}(1-s_\mathrm{f}))}{(1-\bar{d}_\mathrm{f}\,\lambda_\mathrm{f})^2}p_\mathrm{female} > 0. \tag{A5}$$

Substituting $p_\mathrm{controller}$ and $p_\mathrm{female}$ in (A5) with with $p_\mathrm{I}$ and $p_\mathrm{x}$ (see *2.2. Consanguinity and relatedness*) and dividing by $p_\mathrm{I}$ to obtain $r_\mathrm{female}$, yields the condition for increase of the male dispersal gene when the focal male controls his phenotype (condition (1) in the main text).

### *2.2 Consanguinity and relatedness*

The coefficient of consanguinity $p_\mathrm{A,B}$ between two individuals A and B is defined as the probability that a gene drawn at random from a given locus in individual A is identical-by-descent to a gene drawn at random from the same locus in individual B [75]. The second individual may coincide with the first (B=A), in which case we obtain the consanguinity of an individual to itself $p_\mathrm{A,A}$. In particular, the consanguinity of a diploid individual to itself is $p_\mathrm{I} = (1 + f)/2$, where $f$ is the inbreeding coefficient. This is the consanguinity between individuals of opposite sex (mating partners) in a post-competition group and is given by $f = \varphi_\mathrm{MF}\, p_\mathrm{x}$, where $\varphi_\mathrm{MF} = (1 - \bar{a}\,\bar{\omega}\,(2s_\mathrm{m}s_\mathrm{f} - s_\mathrm{f} - s_\mathrm{m})(1 - m_\mathrm{f})\,(1 - m_\mathrm{m})$ expresses the probability that two adults of opposite sex in the same post-competition group were born in the same group. Analogously, the consanguinities of two adult females and two adult males in a post-competition group are $p_{FF} = \varphi_\mathrm{FF}\, p_\mathrm{x}$ and $p_{MM} = \varphi_\mathrm{MM}\, p_\mathrm{x}$, where $\varphi_\mathrm{FF} = (1 - 2\,\bar{a}\,\bar{\omega}\,s_\mathrm{f}(1 - s_\mathrm{f}))(1 - m_\mathrm{f})^2$ and $\varphi_\mathrm{MM} = (1 - 2\,\bar{a}\,\bar{\omega}\,s_\mathrm{m}(1$

$-s_m))(1-m_m)^2$ express the probability that two adult females and two adult males, respectively, in the same post-competition group were born in the same group. $p_x$ is the consanguinity of individuals born in the same group and is equal to:

$$p_x = \frac{1}{4}\left(\frac{1}{N_m}p_I + \frac{N_m-1}{N_m}p_{MM}\right) + \frac{1}{2}f + \frac{1}{4}\left(\frac{1}{N_f}p_I + \frac{N_f-1}{N_f}p_{FF}\right). \tag{A6}$$

Substituting the appropriate consanguinities in the equation above and solving for $p_x$, an expression only dependent on demographic parameters is obtained:

$$p_x = \frac{(N_f+N_m)\,\varphi_{MF}}{8\,N_f\,N_m - 2N_f(N_m-1)\,\varphi_{MM} - 2N_m(N_f-1)\,\varphi_{FF} - (4N_f\,N_m + N_m + N_f)\varphi_{MF}}. \tag{A7}$$

The coefficient of relatedness $r_{A,B}$ between two individuals A and B is defined as the ratio of the consanguinity of individual B to individual A, $p_{A,B}$, and the consanguinity of individual B to herself, $p_{B,B}$ [75]. The relatedness between a focal subadult female and a random female in her group is given by $r_{female} = p_x/p_I$, and coincides with the relatedness between a focal subadult male in his group, $r_{male}$ (see conditions (1-2)). In explicit form:

$$r_{female} = r_{male} = \frac{2(N_f+N_m)}{8N_fN_m - 2N_f(N_m-1)\varphi_{MM} - 2N_m(N_f-1)\varphi_{FF} - 4N_fN_m\varphi_{MF}}. \tag{A8}$$

## 3 Male dispersal

### *3.1 Marginal fitness*

Now, consider that the locus $\mathcal{G}$ controls male dispersal (the phenotype is exhibited by males, but not by females). Assuming vanishing genetic variation, the direction of natural selection is given by Eq. (A3) with all derivatives evaluated at $g = g_m = g_f = \bar{G}$.

The derivative $dW_f/dg_f$ (in Eq. A3) describes how the genic value of a gene drawn from a subadult female impacts on her relative fitness. Since females carry the gene, but do not express it, and competition occurs within sexes, there is no impact on female fitness and this derivative is null.

The derivative $dW_m/dg_m$ (in Eq. A3) describes how the genic value of a gene drawn from a subadult male impacts on his relative fitness. It depends on i) the association between the dispersal phenotype of the focal male and his own relative fitness, the association between the breeding value for the dispersal gene of the male's controller and the focal male's dispersal phenotype, the association between the genic value of the focal male and the breeding value of his controller, and ii) the association between the dispersal phenotype of a random male in the focal group and the focal male's relative fitness, the association between the breeding value for the dispersal gene of the random male's controller and the random male's dispersal phenotype, the association between the genic value of the focal male and the breeding value of the random male's controller. This can be expressed mathematically:

$$\frac{dW_m}{dg_m} = \frac{\partial W_m}{\partial d_m}\frac{dd_m}{dG}\frac{dG}{dg_m} + \frac{\partial W_m}{\partial d_m'}\frac{dd_m'}{dG'}\frac{dG'}{dg_m} = \left(\frac{\partial W_m}{\partial d_m} p_{controller} + \frac{\partial W_m}{\partial d_m'} p_{male}\right)\gamma\,, \tag{A9}$$

where $p_{controller} = dG/dg_m$ is the consanguinity between the focal male and his controller, and $p_{male} = dG'/dg_m$ is the consanguinity between the controller of the focal male and a random male in the focal male's group.

The condition for an increase in population average female dispersal is $dW/dg > 0$ and, as $c_f = c_m = 1/2$ under diploid inheritance, this condition is equivalent to:

$$-\frac{\lambda_m}{1-\bar{d}_m\,\lambda_m} p_{controller} + \frac{(1-\bar{d}_m)(1-2\bar{a}\bar{\omega}s_m(1-s_m))}{(1-\bar{d}_m\,\lambda_m)^2} p_{male} > 0. \tag{A10}$$

Substituting $p_{controller}$ and $p_{male}$ in (A10) with with $p_I$ and $p_x$ (see *2.2. Consanguinity and relatedness*) and dividing by $p_I$ to obtain $r_{male}$, yields the condition for increase of the male dispersal gene when the focal male controls his phenotype (condition (2) in the main text).

## 4 Sex-biased dispersal and migration

### *4.1 Conditions for sex-biased dispersal*

Here we demonstrate that $M_f < M_m$ is a sufficient condition for $m_f^* \geq m_m^*$ and that $M_f > M_m$ is a sufficient condition for $m_f^* \leq m_m^*$, when $\lambda_f = \lambda_m = \lambda$; that is, when the costs of dispersal are equal for the two sexes, greater paternal admixture results in female migration being greater than or equal to male migration, and greater maternal admixture results in male migration being greater than or equal to female migration. Under this assumption and considering that $r_{female} = r_{male} = r$, the marginal fitness function for female dispersal is $\theta_f = -\lambda + (1 - 2\bar{a}\bar{\omega}M_f)(1 - m_f)\, r$ and the marginal fitness function for male dispersal is $\theta_m = -\lambda + (1 - 2\bar{a}\bar{\omega}M_m)(1 - m_m)\, r$. Each migration term can assume either boundary (0 and 1) or intermediate values. Therefore, nine cases are possible. We consider them in turn:

i) $m_f^* = 0$ and $m_m^* = 0$; then $\theta_f|_{m_f=m_f^*,m_m=m_m^*} \leq 0$ and $\theta_m|_{m_f=m_f^*,m_m=m_m^*} = 0$, which requires $M_f \geq (r - \lambda)/(2\bar{a}\bar{\omega} r)$ and $M_m \geq (r - \lambda)/(2\bar{a}\bar{\omega} r)$. Therefore: it is sufficient to have $M_f > M_m$ or $M_f < M_m$ for $m_f^* = m_m^* = 0$.

ii) $m_f^* = 0$ and $0 < m_m^* < 1$; then $\theta_f|_{m_f=m_f^*,m_m=m_m^*} \leq 0$ and $\theta_m|_{m_f=m_f^*,m_m=m_m^*} = 0$, which requires $M_f \geq (r - \lambda)/(2\bar{a}\bar{\omega} r)$ and $M_m < (r - \lambda)/(2\bar{a}\bar{\omega} r)$. Therefore: $M_f > M_m$.

iii) $m_f^* = 0$ and $m_m^* = 1$; then $\theta_m|_{m_f=m_f^*,m_m=m_m^*} \geq 0$, which results in $-\lambda \geq 0$. This contradicts our assumption that $\lambda > 0$ and therefore this case is impossible.

iv) $0 < m_f^* < 1$ and $m_m^* = 0$; then $\theta_f|_{m_f=m_f^*, m_m=m_m^*} = 0$ and $\theta_m|_{m_f=m_f^*, m_m=m_m^*} \leq 0$, which requires $M_f < (r - \lambda)/(2\bar{a}\bar{\omega} r)$ and $M_m \geq (r - \lambda)/(2\bar{a}\bar{\omega} r)$. Therefore: $M_f < M_m$.

v) $0 < m_f^* < 1$ and $0 < m_m^* < 1$; then $\theta_f|_{m_f=m_f^*, m_m=m_m^*} = 0$ and $\theta_m|_{m_f=m_f^*, m_m=m_m^*} = 0$, which requires $m_f^* = 1 - \lambda/(1 - 2\bar{a}\bar{\omega} M_f)r$ and $m_m^* = 1 - \lambda/(1 - 2\bar{a}\bar{\omega} M_m)r$. Therefore: $m_f^* > m_m^*$ if and only if $M_f < M_m$ and $m_f^* < m_m^*$ if and only if $M_f > M_m$.

vi) $0 < m_f^* < 1$ and $m_m^* = 1$; this case is impossible (see case iii)).

vii) $m_f^* = 1$ and $m_m^* = 0$; then $\theta_m|_{m_f=m_f^*, m_m=m_m^*} \geq 0$, which results in $-\lambda \geq 0$. This contradicts our assumption that $0 < \lambda \leq 1$ and therefore this case is impossible.

viii) $m_f^* = 1$ and $0 < m_m^* < 1$; this case is impossible (see case vii)).

ix) $m_f^* = 1$ and $m_m^* = 1$; this case is impossible (see case vii)).

As $M_f < M_m$ obtains in every scenario in which $m_f^* \geq m_m^*$, and as $M_f > M_m$ obtains in every scenario in which $m_f^* \leq m_m^*$, we have demonstrated that $M_f < M_m$ is a sufficient condition for $m_f^* \geq m_m^*$ and $M_f \geq M_m$ is a sufficient condition for $m_f^* \leq m_m^*$, when $\lambda_f = \lambda_m = \lambda$.

Noting that:

$$m_f \geq m_m \Longrightarrow \frac{d_f(1-\lambda)}{1-d_f\lambda} \geq \frac{d_m(1-\lambda)}{1-d_m\lambda} \Longrightarrow d_f \geq d_m, \tag{A11}$$

and

$$m_f \leq m_m \Longrightarrow \frac{d_f(1-\lambda)}{1-d_f\lambda} \leq \frac{d_m(1-\lambda)}{1-d_m\lambda} \Longrightarrow d_f \leq d_m, \tag{A12}$$

it is also true that that $M_f < M_m$ is a sufficient condition for $d_f^* \geq d_m^*$ and that $M_f > M_m$ is a sufficient condition for $d_f^* \leq d_m^*$, when $\lambda_f = \lambda_m = \lambda$.

Secondly, we demonstrate that $\lambda_f < \lambda_m$ is a sufficient condition for $m_f^* \geq m_m^*$ and that $\lambda_f > \lambda_m$ is a sufficient condition for $m_f^* \leq m_m^*$, when $M_f = M_m = M$; that is, when maternal and paternal admixture are equal, greater cost of male dispersal results in female migration being greater or equal to male migration, and greater cost of female dispersal results in male migration being greater or equal to female migration. Under this assumption the marginal fitness function for female dispersal is $\theta_f = -\lambda_f + (1 - 2\bar{a}\bar{\omega}M)(1 - m_f)\, r$ and the marginal fitness function for male dispersal is $\theta_m = -\lambda_m + (1 - 2\bar{a}\bar{\omega}M)(1 - m_m)\, r$. We consider all possible cases.

Analogously to the previous demonstration, cases iii), vi), vii), viii), and ix) lead to a contradiction. We consider the remaining cases:

i) $m_f^* = 0$ and $m_m^* = 0$; then $\theta_f|_{m_f=m_f^*,m_m=m_m^*} \leq 0$ and $\theta_m|_{m_f=m_f^*,m_m=m_m^*} \leq 0$, which requires $\lambda_f \geq (1-2\bar{a}\bar{\omega}M)r$ and $\lambda_m \geq (1-2\bar{a}\bar{\omega}M)r$. Therefore: it is sufficient to have $\lambda_f > \lambda_m$ or $\lambda_f < \lambda_m$ for $m_f^* = m_m^* = 0$.

ii) $m_f^* = 0$ and $0 < m_m^* < 1$; then $\theta_f|_{m_f=m_f^*,m_m=m_m^*} \leq 0$ and $\theta_m|_{m_f=m_f^*,m_m=m_m^*} = 0$, which requires $\lambda_f \geq (1 - 2\bar{a}\bar{\omega}M)r$ and $\lambda_m < (1 - 2\bar{a}\bar{\omega}M)r$. Therefore: $\lambda_f > \lambda_m$.

iv) $0 < m_f^* < 1$ and $m_m^* = 0$; then $\theta_f|_{m_f=m_f^*,m_m=m_m^*} = 0$ and $\theta_m|_{m_f=m_f^*,m_m=m_m^*} \leq 0$, which requires $\lambda_f < (1 - 2\bar{a}\bar{\omega}M)r$ and $\lambda_m \geq (1 - 2\bar{a}\bar{\omega}M)r$. Therefore: $\lambda_f < \lambda_m$.

v) $0 < m_f^* < 1$ and $0 < m_m^* < 1$; then $\theta_f|_{m_f=m_f^*,m_m=m_m^*} = 0$ and $\theta_m|_{m_f=m_f^*,m_m=m_m^*} = 0$, which requires $m_f^* = 1 - \lambda_f/(1 - 2\bar{a}\bar{\omega}M)r$ and $m_m^* = 1 - \lambda_m/(1 - 2\bar{a}\bar{\omega}M)r$. Therefore: $m_f^* > m_m^*$ if and only if $\lambda_f < \lambda_m$ and $m_f^* < m_m^*$ if and only if $\lambda_f > \lambda_m$.

As $\lambda_f < \lambda_m$ obtains in every scenario in which $m_f^* \geq m_m^*$, and as $\lambda_f > \lambda_m$ obtains in every scenario in which $m_f^* \leq m_m^*$, we have demonstrated that $\lambda_f < \lambda_m$ is a sufficient condition for $m_f^* \geq m_m^*$ and $\lambda_f > \lambda_m$ is a sufficient condition for $m_f^* \leq m_m^*$, when $M_f = M_m = M$.

We now demonstrate that it is also true that $\lambda_f \leq \lambda_m$ is a sufficient condition for $d_f^* \geq d_m^*$ and that $\lambda_f \geq \lambda_m$ is a sufficient condition for $d_f^* \leq d_m^*$, when $M_f = M_m = M$. the marginal fitness function for female dispersal is $\theta_f = -\lambda_f + (1-d_f)(1-2\bar{a}\bar{\omega}M)r/(1-d_f\,\lambda_f)$ and the marginal fitness function for male dispersal is $\theta_m = -\lambda_m + (1-d_m)(1-2\bar{a}\bar{\omega}M)r/(1-d_m\,\lambda_m)$. We consider all possible cases. Analogously to the previous demonstration, cases iii), vi), vii), viii), and ix) lead to a contradiction. We consider the remaining cases:

i) $d_f^* = 0$ and $d_m^* = 0$; then $\theta_f|_{d_f=d_f^*,d_m=d_m^*} \leq 0$ and $\theta_m|_{d_f=d_f^*,d_m=d_m^*} \leq 0$, which requires $\lambda_f \geq (1-2\bar{a}\bar{\omega}M)r$ and $\lambda_m \geq (1-2\bar{a}\bar{\omega}M)r$. Therefore: it is sufficient to have $\lambda_f > \lambda_m$, $\lambda_f < \lambda_m$ or $\lambda_f = \lambda_m$ for $d_f^* = d_m^* = 0$.

ii) $d_f^* = 0$ and $0 < d_m^* < 1$; then $\theta_f|_{d_f=d_f^*,d_m=d_m^*} \leq 0$ and $\theta_m|_{d_f=d_f^*,d_m=d_m^*} = 0$, which requires $\lambda_f \geq (1-2\bar{a}\bar{\omega}M)r$ and $(1-2\bar{a}\bar{\omega}M)r = \lambda_m(1-d_m^*\,\lambda_m)/(1-d_m^*)$. Since $(1-d_m^*\,\lambda_m)/(1-d_m^*) \geq 1$, this implies $(1-2\bar{a}\bar{\omega}M)r \geq \lambda_m$. Therefore: $\lambda_f \geq \lambda_m$.

iv) $0 < d_f^* < 1$ and $d_m^* = 0$; then $\theta_f|_{d_f=d_f^*,d_m=d_m^*} = 0$ and $\theta_m|_{d_f=d_f^*,d_m=d_m^*} \leq 0$, which requires $(1-2\bar{a}\bar{\omega}M)r = \lambda_f(1-d_f^*\,\lambda_f)/(1-d_f^*)$ and $\lambda_m \geq (1-2\bar{a}\bar{\omega}M)r$. Since $(1-d_f^*\,\lambda_f)/(1-d_f^*) \geq 1$, this implies $(1-2\bar{a}\bar{\omega}M)r \geq \lambda_f$. Therefore: $\lambda_f \leq \lambda_m$.

v) $0 < d_f^* < 1$ and $0 < d_m^* < 1$; then $\theta_f|_{d_f=d_f^*, d_m=d_m^*} = 0$ and $\theta_m|_{d_f=d_f^*, d_m=d_m^*} = 0$, which requires $d_f^* = ((1-2\bar{a}\bar{\omega}M)r - \lambda_f)/((1-2\bar{a}\bar{\omega}M)r - \lambda_f^2)$ and $d_m^* = ((1-2\bar{a}\bar{\omega}M)r - \lambda_m)/((1-2\bar{a}\bar{\omega}M)r - \lambda_m^2)$. In addition, $\theta_f|_{d_f=d_f^*, d_m=d_m^*} = 0$ also requires $(1-2\bar{a}\bar{\omega}M)r(1-m_f^*) = \lambda_f \Rightarrow (1-2\bar{a}\bar{\omega}M)r > \lambda_f \Rightarrow (1-2\bar{a}\bar{\omega}M)r > \lambda_f^2$. By symmetry: $(1-2\bar{a}\bar{\omega}M)r(1-m_m^*) = \lambda_m \Rightarrow (1-2\bar{a}\bar{\omega}M)r > \lambda_m \Rightarrow (1-2\bar{a}\bar{\omega}M)r > \lambda_m^2$. As a consequence: $((1-2\bar{a}\bar{\omega}M)r - \lambda)/((1-2\bar{a}\bar{\omega}M)r - \lambda^2)$ is a monotonically decreasing function of $\lambda$ for all $0 \le \lambda < (1-2\bar{a}\bar{\omega}M)r \le 1$. Therefore: $d_f^* > d_m^*$ if and only if $\lambda_f < \lambda_m$, and $d_f^* < d_m^*$ if and only if $\lambda_f > \lambda_m$.

*4.2 Convergence stability of migration rates*

Here we demonstrate that it is always possible to find a pair of values of female and male costs of dispersal ($\lambda_f$, $\lambda_m$) such that any given pair of female and male migration rates ($m_f$, $m_m$) are convergence stable [37,45] for any given values of $\bar{a}$, $\bar{\omega}$, $M_f$, $M_m$, $r$. The marginal fitness function for female dispersal is $\theta_f = -\lambda_f + (1-2\bar{a}\bar{\omega}M_f)(1-m_f)\,r$ and the marginal fitness function for male dispersal is $\theta_m = -\lambda_m + (1-2\bar{a}\bar{\omega}M_m)(1-m_m)\,r$. Each migration term can assume either boundary (0 and 1) or intermediate values. Therefore, nine cases are possible. We consider them in turn:

i) $m_f^* = 0$ and $m_m^* = 0$; then $\theta_f|_{m_f=m_f^*, m_m=m_m^*} \le 0$ and $\theta_m|_{m_f=m_f^*, m_m=m_m^*} \le 0$, which requires $\lambda_f \ge (1-2\bar{a}\bar{\omega}M_f)r$ and $\lambda_m \ge (1-2\bar{a}\bar{\omega}M_m)r$. Since $0 \le M_f, M_m \le 0.25$, it follows that $0 \le (1-2\bar{a}\bar{\omega}M_f)r, (1-2\bar{a}\bar{\omega}M_m)r \le 1$. Therefore, we can always achieve $m_f^* = 0$ and $m_m^* = 0$ by setting $\lambda_f = 1$ and $\lambda_m = 1$.

ii) $m_f^* = 0$ and $0 < m_m^* < 1$; then $\theta_f|_{m_f=m_f^*, m_m=m_m^*} \le 0$ and $\theta_m|_{m_f=m_f^*, m_m=m_m^*} = 0$, which requires $\lambda_f \ge (1-2\bar{a}\bar{\omega}M_f)r$ and $\lambda_m = (1-m_m^*)(1-2\bar{a}\bar{\omega}M_m)r$. Since $0 \le (1-m_m^*)(1-2\bar{a}\bar{\omega}M_m)r \le 1$, we can achieve $m_f^* = 0$ and $0 < m_m^* < 1$ by setting $\lambda_f = 1$ and $\lambda_m = (1-m_m^*)(1-2\bar{a}\bar{\omega}M_m)r$.

iii) $m_f^* = 0$ and $m_m^* = 1$; then $\theta_f|_{m_f=m_f^*, m_m=m_m^*} \le 0$ and $\theta_m|_{m_f=m_f^*, m_m=m_m^*} \ge 0$, which requires $\lambda_f \ge (1-2\bar{a}\bar{\omega}M_f)r$ and $\lambda_m = 0$. Therefore, we can achieve $m_f^* = 0$ and $m_m^* = 1$ by setting $\lambda_f = 1$ and $\lambda_m = 0$.

iv) $0 < m_f^* < 1$ and $m_m^* = 0$; then $\theta_f|_{m_f=m_f^*, m_m=m_m^*} = 0$ and $\theta_m|_{m_f=m_f^*, m_m=m_m^*} \le 0$, which requires $\lambda_f = (1-m_f^*)(1-2\bar{a}\bar{\omega}M_f)r$ and $\lambda_m \ge (1-2\bar{a}\bar{\omega}M_m)r$. Since $0 \le (1-m_f^*)(1-2\bar{a}\bar{\omega}M_f)r \le 1$, we can achieve $0 < m_f^* < 1$ and $m_m^* = 0$ by setting $\lambda_f = (1-m_f^*)(1-2\bar{a}\bar{\omega}M_f)r$ and $\lambda_m = 1$.

v) $0 < m_f^* < 1$ and $0 < m_m^* < 1$; then $\theta_f|_{m_f=m_f^*, m_m=m_m^*} = 0$ and $\theta_m|_{m_f=m_f^*, m_m=m_m^*} = 0$, which requires $\lambda_f = (1 - m_f^*)(1 - 2\ \bar{a}\ \bar{\omega}\ M_f)r$ and $\lambda_m = (1 - m_m^*)(1 - 2\ \bar{a}\ \bar{\omega}\ M_m)r$.

vi) $0 < m_f^* < 1$ and $m_m^* = 1$; then $\theta_f|_{m_f=m_f^*, m_m=m_m^*} = 0$ and $\theta_m|_{m_f=m_f^*, m_m=m_m^*} \geq 0$, which requires $\lambda_f = (1 - m_f^*)(1 - 2\ \bar{a}\ \bar{\omega}\ M_f)r$ and $\lambda_m = 0$.

vii) $m_f^* = 1$ and $m_m^* = 0$; then $\theta_f|_{m_f=m_f^*, m_m=m_m^*} \geq 0$ and $\theta_m|_{m_f=m_f^*, m_m=m_m^*} \leq 0$, which requires $\lambda_f = 0$ and $\lambda_m \geq (1 - 2\ \bar{a}\ \bar{\omega}\ M_m)r$.

viii) $m_f^* = 1$ and $0 < m_m^* < 1$; then $\theta_f|_{m_f=m_f^*, m_m=m_m^*} \geq 0$ and $\theta_m|_{m_f=m_f^*, m_m=m_m^*} = 0$, which requires $\lambda_f = 0$ and $\lambda_m = (1 - m_m^*)(1 - 2\ \bar{a}\ \bar{\omega}\ M_m)r$.

ix) $m_f^* = 1$ and $m_m^* = 1$; then $\theta_f|_{m_f=m_f^*, m_m=m_m^*} \geq 0$ and $\theta_m|_{m_f=m_f^*, m_m=m_m^*} \geq 0$, which requires $\lambda_f = 0$ and $\lambda_m = 0$.

Therefore, in every case, there exist values in the range $0 \leq \lambda_f, \lambda_m \leq 1$ that satisfy these requirements, and hence any desired $m_f^*$ and $m_m^*$ may be obtained, irrespective of the values of $\bar{a}$, $\bar{\omega}$, $M_f$, $M_m$, $r$, by choosing appropriate values of $\lambda_f$ and $\lambda_m$.

*4.3 Individual-based simulations for dispersal evolution*

We develop an individual-based simulation model of dispersal evolution for the purpose of illustration and to assess the robustness of our analytical results. We consider a population of $p$ = 400 groups, each containing $N_f$ = 10 adult females and $N_m$ =10 adult males. Each individual carries two loci, one controlling female dispersal and the other controlling male dispersal; each locus has two alleles, each of which is represented by a real-valued number, multiple of 0.02, lying between 0 and 1, including the extremes. At initialisation, all allele values are assigned a randomly-chosen allowed value. Each adult female produces $k$ = 100 daughters and $k$ = 100 sons, mating randomly each time, so that the offspring of the same mother do not always share the same father (absolute promiscuity). When each offspring is produced, each gene mutates with probability $M$ = 0.001 to a new value, which can be the current value, the current value + 0.02, or the current value – 0.02, with equal probability. Subadult females disperse from the natal group with a probability determined by the average of her female dispersal alleles and reach a randomly-selected group with probability $1 - \lambda_f$; subadult males disperse from the natal group with a probability determined by the average of his male dispersal alleles and reach a randomly-selected group with probability $1- \lambda_m$. Warfare is modelled as follows: for tractability, we imagine that all groups are arranged in a circle (at every generation the order of groups is randomised, to avoid neighbour effects); each group attacks the next one in the circle clockwise and wins the war with probability $\omega$ = 0.5; each individual is assigned a competitiveness value in

their own group (“home”), which depends on whether the group has been attacked and which group won, and a competitiveness value in the neighbouring group (“abroad”), which depends on whether the focal group has attacked that group and which group won (see Methods). During the following phase (density dependent regulation), $N_f = 10$ females are randomly sampled (using competitiveness values as weights) to become adults; analogously $N_m = 10$ males are randomly sampled (using competitiveness values as weights) to become adults. These then produce the next generation of subadults in the following cycle. We track $G = 10{,}000$ generations of evolution and calculate average allelic values of both male and female dispersal genes in each generation. The data points shown in Figure A4.3.1 come from a single simulation and are the mean of the average allelic values of the last 1000 generations of evolution. The simulation code is provided in a *Wolfram Mathematica* file.

Simulation results show a reasonable fit with our analytical predictions (Fig A4.3.1) and the evolutionary trajectories followed are the ones expected from vector field plots (Fig. A4.3.2-A.4.3.3). The small discrepancies between the simulation data and corresponding analytical predictions owe to random drift and spontaneous mutation, neither of which are considered in our mathematical analysis.

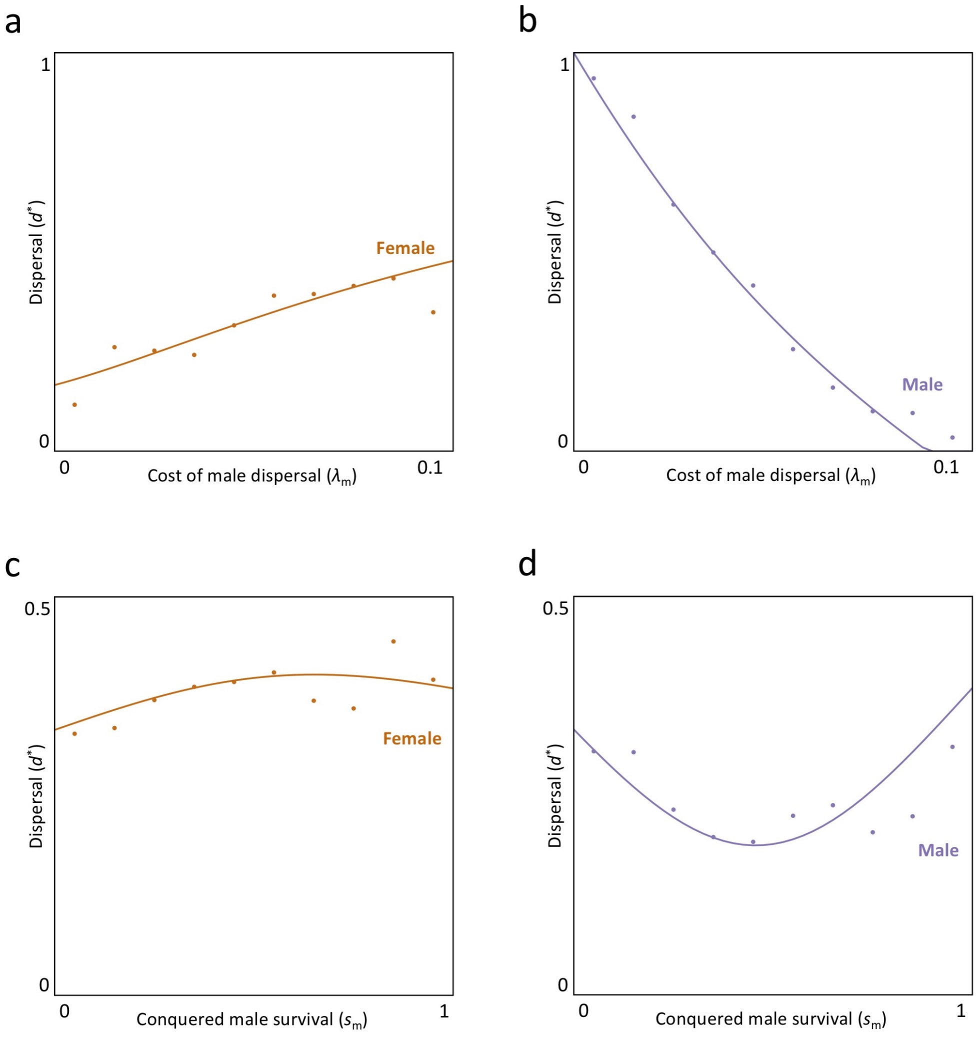


**Figure A4.3.1 – Evolution of sex-biased dispersal and migration.** Analytical predictions (lines) and individual-based simulation results (filled circles) for convergence-stable levels of female dispersal ($d_f^*$, orange) and male dispersal ($d_m^*$, purple) as a function of cost of male dispersal ($\lambda_m$; panels a-b; other parameter values are $\lambda_f = 0.05$, $s_f = 1$, $s_m = 0$, $N_f = N_m = 10$, $\bar{a} = 1$, $\bar{\omega} = 0.5$) and the probability that a conquered male obtains a breeding spot ($s_m$; panels c-d; other parameter values are $\lambda_f = \lambda_m = 0.05$, $s_f = 1$, $N_f = N_m = 10$, $\bar{a} = 1$, $\bar{\omega} = 0.5$).

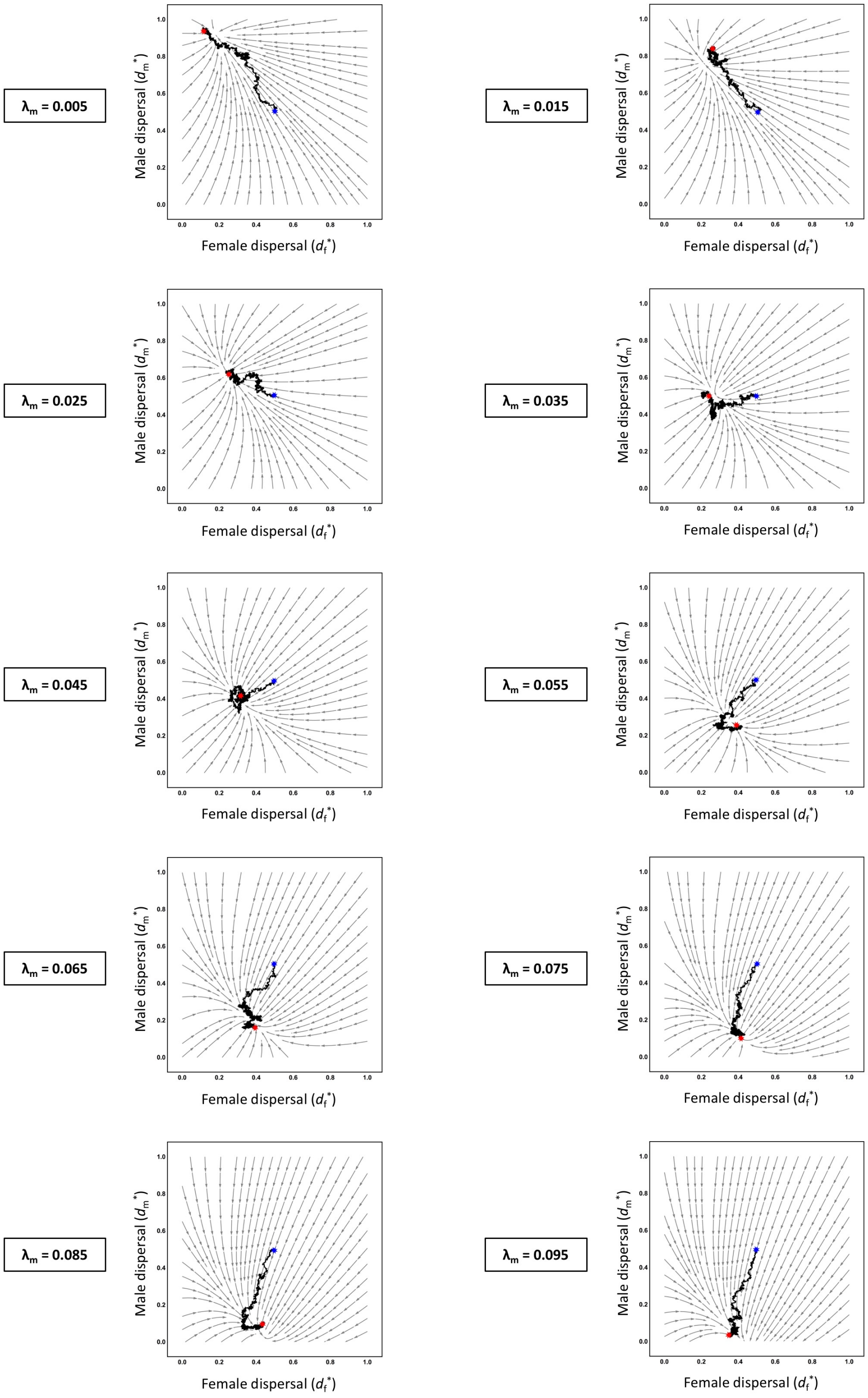


**Figure A4.3.2 – Dispersal evolution with varying male costs of dispersal.** Vector field plots and evolutionary trajectories of female dispersal ($d_f^*$) and male dispersal ($d_m^*$) resulting from individual-based simulations, for different values of male cost of dispersal $\lambda_m$. Blue star indicates average allelic values at the start of the simulation; black dots indicate average allelic value in each successive generation; red star indicates average allelic values in the last generation. Parameter values: $\lambda_f = 0.05$, $s_f = 1$, $s_m = 0$, $N_f = N_m = 10$, $\bar{a} = 1$, $\bar{\omega} = 0.5$.

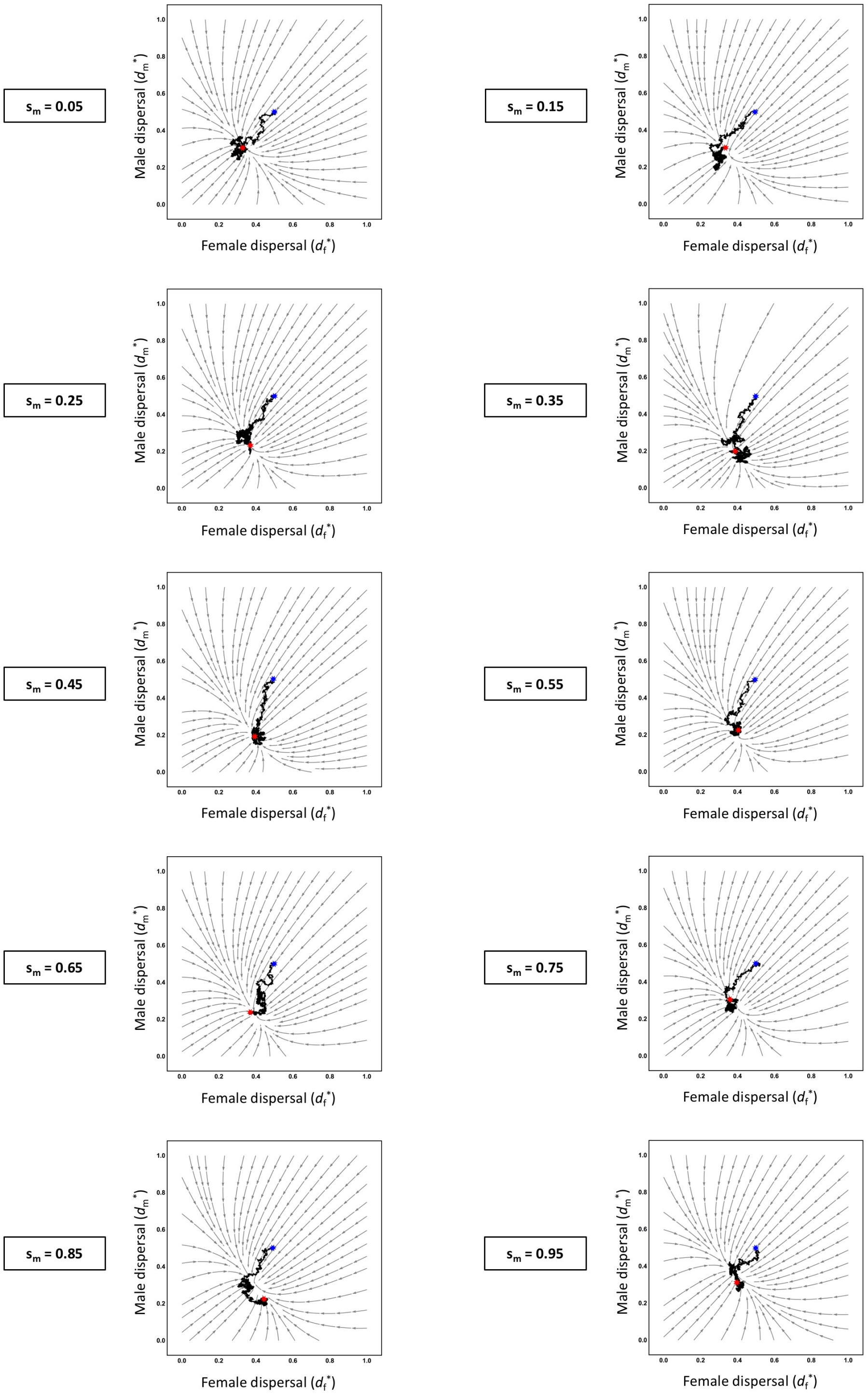


**Figure A4.3.3 – Dispersal evolution with varying conquered male survival.** Vector field plots and evolutionary trajectories of female dispersal ($d_f^*$) and male dispersal ($d_m^*$) resulting from individual-based simulations, for different values of conquered male survival $s_m$. Blue star indicates average allelic values at the start of the simulation; black dots indicate average allelic value in each successive generation; red star indicates average allelic values in the last generation. Parameter values: $\lambda_f = \lambda_m = 0.05$, $s_f = 1$, $N_f = N_m = 10$, $\bar{a} = 1$, $\bar{\omega} = 0.5$.

## 5 Belligerence

### *5.1 Marginal fitness*

Now, consider that the locus $\mathcal{G}$ controls belligerence (the phenotype is exhibited only by males). Assuming vanishing genetic variation, the direction of natural selection is given by Eq. (A3) with all derivatives evaluated at $g = g_{\mathrm{m}} = g_{\mathrm{f}} = \bar{G}$.

The derivative $\mathrm{d}W_{\mathrm{f}}/\mathrm{d}g_{\mathrm{f}}$ (in Eq. A3) describes how the genic value of a gene drawn from a subadult female impacts on her relative fitness. Since females carry the gene, but do not express it, only indirect fitness is considered. This depends on the association between the belligerence phenotype of a male randomly sampled from the focal group and the focal female's relative fitness, the association between the breeding value of that male's controller and that male's belligerence phenotype, and the association between the breeding value of that male's controller and the focal female's genic value. This can be expressed mathematically:

$$\frac{\mathrm{d}W_{\mathrm{f}}}{\mathrm{d}g_{\mathrm{f}}} = \frac{\partial W_{\mathrm{f}}}{\partial A'}\frac{\mathrm{d}A'}{\mathrm{d}G'}\frac{\mathrm{d}G'}{\mathrm{d}g_{\mathrm{f}}} = \frac{\partial W_{\mathrm{f}}}{\partial A'}\, p_{\mathrm{female|controller}}\, \gamma \,, \qquad \text{(A13)}$$

where $p_{\mathrm{female|controller}} = \mathrm{d}G'/\mathrm{d}g_{\mathrm{f}}$ is the consanguinity between the focal female and the controller of a random male in her group, and $\gamma = \mathrm{d}A'/\mathrm{d}G'$ is again the association between an individual's phenotype and its controller's breeding value.

The derivative $\mathrm{d}W_{\mathrm{m}}/\mathrm{d}g_{\mathrm{m}}$ (in Eq. A3) describes how the genic value of a gene drawn from a subadult male impacts on his relative fitness. It depends on i) the association between the belligerence phenotype of the focal male and his own relative fitness, the association between the breeding value for the belligerence gene of the male's controller and the focal male's belligerence phenotype, the association between the genic value of the focal male and the breeding value of his controller, and ii) the association between the belligerence phenotype of a random male in the focal group and the focal male's relative fitness, the association between the breeding value for the belligerence gene of the random male's controller and the random male's belligerence phenotype, the association between the genic value of the focal male and the breeding value of the random male's controller. This can be expressed mathematically:

$$\frac{\mathrm{d}W_{\mathrm{m}}}{\mathrm{d}g_{\mathrm{m}}} = \frac{\partial W_{\mathrm{m}}}{\partial A}\frac{\mathrm{d}A}{\mathrm{d}G}\frac{\mathrm{d}G}{\mathrm{d}g_{\mathrm{m}}} + \frac{\partial W_{\mathrm{m}}}{\partial A'}\frac{\mathrm{d}A'}{\mathrm{d}G'}\frac{\mathrm{d}G'}{\mathrm{d}g_{\mathrm{m}}} = \left(\frac{\partial W_{\mathrm{m}}}{\partial A}\, p_{\mathrm{focal|controller}} + \frac{\partial W_{\mathrm{m}}}{\partial A'}\, p_{\mathrm{male|controller}}\right)\gamma \,, \qquad \text{(A14)}$$

where $p_{\mathrm{focal|controller}} = \mathrm{d}G/\mathrm{d}g_{\mathrm{m}}$ is the consanguinity between the focal male and his controller, $p_{\mathrm{male|controller}} = \mathrm{d}G'/\mathrm{d}g_{\mathrm{m}}$ is the consanguinity between the focal male and the controller of a random male in his group, and $\gamma = \mathrm{d}A'/\mathrm{d}G'$ is the correlation between an individual's phenotype and its controller's breeding value.

Considering that both $a$ and $t$ are functions of $A$, it results that $\partial W_{\mathrm{f}}/\partial A' = (\partial W_{\mathrm{f}}/\partial a')(\partial a'/\partial A')$, $\partial W_{\mathrm{m}}/\partial A = (\partial W_{\mathrm{m}}/\partial t(A))(\partial t(A)/\partial A)$, and $\partial W_{\mathrm{m}}/\partial A' = (\partial W_{\mathrm{m}}/\partial a')(\partial a'/\partial A') + (\partial W_{\mathrm{m}}/\partial t(A'))(\partial t(A')/\partial A')$, where $\partial t(A)/\partial A = \partial t(A')/\partial A' = -\, t(\bar{A})\, \mathrm{c_a}$ and $\partial a'/\partial A' = b_{\mathrm{a}}$. Substituting these expressions and Eq. A12-13 into Eq. A3, we obtain:

$$\frac{\mathrm{d}W}{\mathrm{d}g} = c_\mathrm{f}\frac{\partial W_\mathrm{f}}{\partial a'} b_\mathrm{a}\, p_{\mathrm{female|controller}}\,\gamma + c_\mathrm{m}\left(\frac{\partial W_\mathrm{m}}{\partial t(A)}(-t(\bar{A})\,c_\mathrm{a})\,p_{\mathrm{focal|controller}} + \left(\frac{\partial W_\mathrm{m}}{\partial a'} b_\mathrm{a} + \frac{\partial W_\mathrm{m}}{\partial t(A')}(-t(\bar{A})\,c_\mathrm{a})\right) p_{\mathrm{male|controller}}\right)\gamma\ . \quad \text{(A15)}$$

The condition for an increase in population average female dispersal is $\mathrm{d}W/\mathrm{d}g > 0$ and, as $c_\mathrm{f} = c_\mathrm{m} = 1/2$ under diploid inheritance, this condition is equivalent to:

$$-c_\mathrm{a}\, p_{\mathrm{focal|controller}} + \left(1 - 2\,\bar{a}\,\bar{\omega}\,s_\mathrm{m}\,(1 - s_\mathrm{m})\right) c_\mathrm{a}\, p_{\mathrm{male|controller}} + \bar{\omega}\,(1 - s_\mathrm{m})\,b_\mathrm{a}\, p_{\mathrm{male|controller}} + \bar{\omega}\,(1 - s_\mathrm{f})\,b_\mathrm{a} p_{\mathrm{female|controller}} > 0\ . \quad \text{(A16)}$$

Dividing by $p_{\mathrm{focal|controller}}$ to obtain $R_{\mathrm{male|controller}} = p_{\mathrm{male|controller}}/p_{\mathrm{focal|controller}}$ and $R_{\mathrm{female|controller}} = p_{\mathrm{female|controller}}/p_{\mathrm{focal|controller}}$ (see *5.2. Consanguinity and relatedness*) yields condition (3) in the main text.

Condition (A16) can be rearranged as follows:

$$\frac{c_\mathrm{a}}{b_\mathrm{a}} < \frac{\bar{\omega}(1-m_\mathrm{m})^2\left((1-s_\mathrm{m})\,p_{\mathrm{male|controller}}+(1-s_\mathrm{f})\,p_{\mathrm{female|controller}}\right)}{(1-m_\mathrm{m})^2\,p_{\mathrm{focal|controller}}-\varphi_{\mathrm{MM}}\,p_{\mathrm{male|controller}}}\ , \quad \text{(A17)}$$

where $\varphi_{\mathrm{MM}} = (1 - 2\,\bar{a}\,\bar{\omega}\,s_\mathrm{m}\,(1 - s_\mathrm{m}))(1 - m)^2$ is the probability that two males in a post-competition group were born in the same group. The RHS of (A17) can be conceptualised as "potential for belligerence" (cf. [76]) and is termed $\lambda_\mathrm{A}$.

Substituting $p_{\mathrm{focal|controller}}$, $p_{\mathrm{female|controller}}$, and $p_{\mathrm{male|controller}}$ in (A17) with the appropriate consanguinities (see *5.2. Consanguinity and relatedness*) yields the condition for increase of the belligerence gene. When the phenotype is controlled by the genotype of the father of the focal individual ($p_\mathrm{s}$, $p_{\mathrm{female|faher}}$, $p_{\mathrm{male|father}}$) this is:

$$\frac{c_\mathrm{a}}{b_\mathrm{a}} < \frac{\bar{\omega}(1-m_\mathrm{m})\left((1-s_\mathrm{m})(1-m_\mathrm{m})+(1-s_\mathrm{f})(1-m_\mathrm{f})\right)(4N_\mathrm{m}N_\mathrm{f}-N_\mathrm{f}(N_\mathrm{m}-1)\varphi_{\mathrm{MM}}-N_\mathrm{m}(N_\mathrm{f}-1)-2N_\mathrm{m}N_\mathrm{f}\varphi_{\mathrm{MF}}+(N_\mathrm{m}+N_\mathrm{f})\varphi_{\mathrm{MF}}+(N_\mathrm{m}+N_\mathrm{f})(\varphi_{\mathrm{MF}}+\varphi_{\mathrm{MM}})(N_\mathrm{m}-1)}{(N_\mathrm{m}-\varphi_{\mathrm{MM}})(4N_\mathrm{m}N_\mathrm{f}-N_\mathrm{f}(N_\mathrm{m}-1)\varphi_{\mathrm{MM}}-N_\mathrm{m}(N_\mathrm{f}-1)\varphi_{\mathrm{FF}}-2N_\mathrm{m}N_\mathrm{f}\varphi_{\mathrm{MF}}+(N_\mathrm{m}+N_\mathrm{f})\varphi_{\mathrm{MF}})-\varphi_{\mathrm{MM}}(N_\mathrm{m}+N_\mathrm{f})(\varphi_{\mathrm{MF}}+\varphi_{\mathrm{MM}})(N_\mathrm{m}-1)}\ . \quad \text{(A18)}$$

Setting $m_\mathrm{f} = m_\mathrm{m} = m$ and assuming that $N_\mathrm{f} = N_\mathrm{m} = N$ is large, thus neglecting terms of order $1/N^2$, these reduce to:

$$\frac{c_\mathrm{a}}{b_\mathrm{a}} < \frac{2\bar{\omega}x(1-m)^2\left(1+\frac{1}{2}\bar{a}\,\bar{\omega}(1-m)^2(s_\mathrm{f}-s_\mathrm{m})(1-s_\mathrm{f}-s_\mathrm{m})\right)}{N\left(1-(1-m)^2\left(1-2\bar{a}\bar{\omega}x(1-x)\right)\right)}\ , \quad \text{(A19)}$$

where $x = 1 - (s_\mathrm{m} + s_\mathrm{f})/2$. Noting that $b_\mathrm{a} = \bar{a}\,B_\mathrm{a} / (1 - m_\mathrm{m})$ and $c_\mathrm{a} = C_\mathrm{a}$ in Lehmann & Feldman's [7] notation, this recovers their result see (see Eq. (2.2) in [50]).

When the phenotype is controlled by the genotype of the mother of the focal individual ($p_{\mathrm{s}}$, $p_{\mathrm{female|mother}}$, $p_{\mathrm{male|mother}}$) the condition is given by:

$$\frac{c_{\mathrm{a}}}{b_{\mathrm{a}}} < \frac{\bar{\omega}(1-m_{\mathrm{m}})\big((1-s_{\mathrm{m}})(1-m_{\mathrm{m}})+(1-s_{\mathrm{f}})(1-m_{\mathrm{f}})\big)(4N_{\mathrm{m}}N_{\mathrm{f}}-N_{\mathrm{f}}(N_{\mathrm{m}}-1)\varphi_{\mathrm{MM}}-N_{\mathrm{m}}(N_{\mathrm{f}}-1)-2N_{\mathrm{m}}N_{\mathrm{f}}\varphi_{\mathrm{MF}}+(N_{\mathrm{m}}+N_{\mathrm{f}})\varphi_{\mathrm{MF}}+(N_{\mathrm{m}}+N_{\mathrm{f}})(\varphi_{\mathrm{MF}}+\varphi_{\mathrm{FF}})(N_{\mathrm{f}}-1)}{(N_{\mathrm{f}}-\varphi_{\mathrm{MM}})(4N_{\mathrm{m}}N_{\mathrm{f}}-N_{\mathrm{f}}(N_{\mathrm{m}}-1)\varphi_{\mathrm{MM}}-N_{\mathrm{m}}(N_{\mathrm{f}}-1)\varphi_{\mathrm{FF}}-2N_{\mathrm{m}}N_{\mathrm{f}}\varphi_{\mathrm{MF}}+(N_{\mathrm{m}}+N_{\mathrm{f}})\varphi_{\mathrm{MF}})-\varphi_{\mathrm{MM}}(N_{\mathrm{m}}+N_{\mathrm{f}})(\varphi_{\mathrm{MF}}+\varphi_{\mathrm{FF}})(N_{\mathrm{f}}-1)} \;. \tag{A20}$$

When the genotype of the focal individual controls the phenotype ($p_{\mathrm{I}}$, $p_{\mathrm{female|individual}}$, $p_{\mathrm{male|individual}}$) this is:

$$\frac{c_{\mathrm{a}}}{b_{\mathrm{a}}} < \frac{2\bar{\omega}(1-m_{\mathrm{m}})\big((1-s_{\mathrm{m}})(1-m_{\mathrm{m}})+(1-s_{\mathrm{f}})(1-m_{\mathrm{f}})\big)(N_{\mathrm{f}}+N_{\mathrm{m}})}{4N_{\mathrm{m}}N_{\mathrm{f}}-N_{\mathrm{f}}(N_{\mathrm{m}}-1)\varphi_{\mathrm{MM}}-N_{\mathrm{m}}(N_{\mathrm{f}}-1)\varphi_{\mathrm{FF}}-2N_{\mathrm{m}}N_{\mathrm{f}}\varphi_{\mathrm{MF}}+(N_{\mathrm{m}}+N_{\mathrm{f}})\varphi_{\mathrm{MF}}} \;. \tag{A21}$$

Finally, granting full control over the phenotype to paternal-origin genes ($p_{\mathrm{I|paternal}}$, $p_{\mathrm{female|paternal}}$, $p_{\mathrm{male|paternal}}$) we have:

$$\frac{c_{\mathrm{a}}}{b_{\mathrm{a}}} < \frac{\bar{\omega}(1-m_{\mathrm{m}})\big((1-s_{\mathrm{m}})(1-m_{\mathrm{m}})+(1-s_{\mathrm{f}})(1-m_{\mathrm{f}})\big)(4N_{\mathrm{m}}N_{\mathrm{f}}-N_{\mathrm{f}}(N_{\mathrm{m}}-1)\varphi_{\mathrm{MM}}-N_{\mathrm{m}}(N_{\mathrm{f}}-1)\varphi_{\mathrm{FF}}+(N_{\mathrm{f}}+N_{\mathrm{m}})(N_{\mathrm{m}}-1)\varphi_{\mathrm{MM}}+N_{\mathrm{m}}(N_{\mathrm{m}}-N_{\mathrm{f}})\varphi_{\mathrm{MF}})}{N_{\mathrm{m}}(8N_{\mathrm{f}}N_{\mathrm{m}}-2N_{\mathrm{f}}(N_{\mathrm{m}}-1)\varphi_{\mathrm{MM}}-2N_{\mathrm{m}}(N_{\mathrm{f}}-1)\varphi_{\mathrm{FF}}-4N_{\mathrm{f}}N_{\mathrm{m}}\varphi_{\mathrm{MF}})-\varphi_{\mathrm{MM}}(4N_{\mathrm{f}}N_{\mathrm{m}}-N_{\mathrm{f}}(N_{\mathrm{m}}-1)\varphi_{\mathrm{MM}}-N_{\mathrm{m}}(N_{\mathrm{f}}-1)\varphi_{\mathrm{FF}}+(N_{\mathrm{f}}+N_{\mathrm{m}})(N_{\mathrm{m}}-1)\varphi_{\mathrm{MM}}+N_{\mathrm{m}}(N_{\mathrm{m}}-N_{\mathrm{f}})\varphi_{\mathrm{MF}})} \;. \tag{A22}$$

and granting full control over the phenotype to paternal-origin genes ($p_{\mathrm{I|maternal}}$, $p_{\mathrm{female|maternal}}$, $p_{\mathrm{male|maternal}}$), we obtain:

$$\frac{c_{\mathrm{a}}}{b_{\mathrm{a}}} < \frac{\bar{\omega}(1-m_{\mathrm{m}})\big((1-s_{\mathrm{m}})(1-m_{\mathrm{m}})+(1-s_{\mathrm{f}})(1-m_{\mathrm{f}})\big)(4N_{\mathrm{m}}N_{\mathrm{f}}-N_{\mathrm{f}}(N_{\mathrm{m}}-1)\varphi_{\mathrm{MM}}-N_{\mathrm{m}}(N_{\mathrm{f}}-1)\varphi_{\mathrm{FF}}+(N_{\mathrm{f}}+N_{\mathrm{m}})(N_{\mathrm{f}}-1)\varphi_{\mathrm{FF}}+N_{\mathrm{f}}(N_{\mathrm{f}}-N_{\mathrm{m}})\varphi_{\mathrm{MF}})}{N_{\mathrm{f}}(8N_{\mathrm{f}}N_{\mathrm{m}}-2N_{\mathrm{f}}(N_{\mathrm{m}}-1)\varphi_{\mathrm{MM}}-2N_{\mathrm{m}}(N_{\mathrm{f}}-1)\varphi_{\mathrm{FF}}-4N_{\mathrm{f}}N_{\mathrm{m}}\varphi_{\mathrm{MF}})-\varphi_{\mathrm{MM}}(4N_{\mathrm{f}}N_{\mathrm{m}}-N_{\mathrm{f}}(N_{\mathrm{m}}-1)\varphi_{\mathrm{MM}}-N_{\mathrm{m}}(N_{\mathrm{f}}-1)\varphi_{\mathrm{FF}}+(N_{\mathrm{f}}+N_{\mathrm{m}})(N_{\mathrm{f}}-1)\varphi_{\mathrm{FF}}+N_{\mathrm{f}}(N_{\mathrm{f}}-N_{\mathrm{m}})\varphi_{\mathrm{MF}})} \;. \tag{A23}$$

### *5.2 Consanguinity and relatedness*

The consanguinity of a focal subadult, independent of sex, to a parent is given by $p_{\mathrm{s}} = ½(½(1+f)) + ½f$ (where $f$ is the inbreeding coefficient). The consanguinity of the focal subadult male to a random subadult male in his post-migration group is equal to the probability that they were born in the same group and that neither migrated, namely $p_{\mathrm{male|individual}} = (1 - m_{\mathrm{m}})^2\, p_{\mathrm{x}}$. The consanguinity of the focal subadult male to a random subadult male in his post-migration group is derived analogously and is given by $p_{\mathrm{female|individual}} = (1 - m_{\mathrm{f}})^2\, p_{\mathrm{x}}$. Substituting (A7) in $p_{\mathrm{male|individual}}$ and $p_{\mathrm{female|individual}}$, we obtain explicit expressions (Table A1). Dividing $p_{\mathrm{male|individual}}$ and $p_{\mathrm{female|individual}}$ by $p_{\mathrm{I}}$ yields relatedness coefficients $R_{\mathrm{male|individual}}$ and $R_{\mathrm{female|individual}}$ in their explicit form (Table A1).

The consanguinity of a focal subadult male to the father of a random subadult male in their post-migration group is given by:

$$p_{\mathrm{male|father}} = (1-m_{\mathrm{m}})^2\left(\frac{1}{N_{\mathrm{m}}}p_{\mathrm{S}} + \frac{(N_{\mathrm{m}}-1)(f+p_{\mathrm{MM}})}{2N_{\mathrm{m}}}\right). \tag{A24}$$

That is, with probability $(1 - m_{\mathrm{m}})^2$ both the focal male and his male groupmate were born in the same group: in this case, with probability $1/N_{\mathrm{m}}$ they share the same father and hence the consanguinity between the focal subadult male and his father is $p_{\mathrm{S}}$; alternatively, with probability $(N_{\mathrm{m}} - 1)/N_{\mathrm{m}}$ the two males do not have the same father and hence the consanguinity of the focal subadult male with the father of the other male is equal to $(f + p_{\mathrm{MM}})/2$. A similar rationale is

followed to obtain the consanguinity of a focal subadult female to the father of a random subadult male in their group:

$$p_{\mathrm{female|father}} = (1 - m_{\mathrm{m}})(1 - m_{\mathrm{f}})\left(\frac{1}{N_i}p_{\mathrm{S}} + \frac{(N_{\mathrm{m}}-1)(f+p_{\mathrm{MM}})}{2N_{\mathrm{m}}}\right). \quad \text{(A25)}$$

The consanguinities of a subadult male and a subadult female to the mother of a random subadult male in the group are derived analogously:

$$p_{\mathrm{male|mother}} = (1 - m_{\mathrm{m}})^2\left(\frac{1}{N_{\mathrm{f}}}p_{\mathrm{S}} + \frac{(N_{\mathrm{f}}-1)(f+p_{\mathrm{FF}})}{2N_{\mathrm{f}}}\right), \quad \text{(A26)}$$

$$p_{\mathrm{female|mother}} = (1 - m_{\mathrm{m}})(1 - m_{\mathrm{f}})\left(\frac{1}{N_{\mathrm{f}}}p_{\mathrm{S}} + \frac{(N_{\mathrm{f}}-1)(f+p_{\mathrm{FF}})}{2N_{\mathrm{f}}}\right). \quad \text{(A27)}$$

Substituting the appropriate consanguinities, including (A7), in the equations above yields $p_{\mathrm{s}}$, $p_{\mathrm{male|father}}$, $p_{\mathrm{female|father}}$, $p_{\mathrm{male|mother}}$, and $p_{\mathrm{female|mother}}$ in their explicit form (Table A2). Dividing $p_{\mathrm{male|father}}$, $p_{\mathrm{female|father}}$, $p_{\mathrm{male|mother}}$, and $p_{\mathrm{female|mother}}$ by $p_{\mathrm{s}}$, yields $R_{\mathrm{male|father}}$, $R_{\mathrm{female|father}}$, $R_{\mathrm{male|mother}}$, and $R_{\mathrm{female|mother}}$, respectively, in their explicit form (Table A2).

The consanguinities of a focal individual to its paternal-origin genes $p_{\mathrm{I|paternal}}$ and to its maternal-origin genes $p_{\mathrm{I|maternal}}$ are both equal to $p_{\mathrm{I}}$, the consanguinity of a focal individual to herself (Table A3). The consanguinity between a focal subadult male and the paternal-origin genes of a random subadult male in his group $p_{\mathrm{male|paternal}}$ is given by

$$p_{\mathrm{male|paternal}} = (1 - m_{\mathrm{m}})^2\left(\frac{1}{2}\left(\frac{1}{N_{\mathrm{m}}}p_{\mathrm{I}} + \frac{N_{\mathrm{m}}-1}{N_{\mathrm{m}}}p_{\mathrm{MM}}\right) + \frac{1}{2}f\right). \quad \text{(A28)}$$

That is with probability $(1 - m_{\mathrm{m}})^2$ both the focal male and his male groupmate were born in the same group. And with probability 1/2, a gene picked from the focal male is the paternal gene: in this case, with probability $1/N_{\mathrm{m}}$, the two males share the same father and so the gene is identical-by-descent to the paternal-origin gene in the random male with probability $p_{\mathrm{I}}$; alternatively, with probability $(N_{\mathrm{m}} - 1)/N_{\mathrm{m}}$, the two males do not share the same father and therefore the probability of identity-by-descent between the gene we have picked and the paternal-origin gene in the random male is $p_{\mathrm{FF}}$. On the other hand, with probability 1/2, a gene picked from the focal male is the maternal gene: in this case, the gene is identical-by-descent to the paternal-origin gene of the random male with probability $f$. The consanguinity between a focal subadult female and the paternal-origin genes of a random subadult male in her group $p_{\mathrm{female|paternal}}$ is derived analogously:

$$p_{\mathrm{female|paternal}} = (1 - m_{\mathrm{m}})(1 - m_{\mathrm{f}})\left(\frac{1}{2}\left(\frac{1}{N_{\mathrm{m}}}p_{\mathrm{I}} + \frac{N_{\mathrm{m}}-1}{N_{\mathrm{m}}}p_{\mathrm{MM}}\right) + \frac{1}{2}f\right). \quad \text{(A29)}$$

Finally, the same rationale is followed to derive the consanguinities of a focal individual to the maternal-origin genes of a random male in its group. These are given by:

$$p_{\text{male|maternal}} = (1 - m_{\text{m}})^2 \left(\frac{1}{2}\left(\frac{1}{N_{\text{f}}} p_{\text{I}} + \frac{N_{\text{f}}-1}{N_{\text{f}}} p_{\text{FF}}\right) + \frac{1}{2} f\right), \quad \text{(A30)}$$

$$p_{\text{male|maternal}} = (1 - m_{\text{m}})(1 - m_{\text{f}}) \left(\frac{1}{2}\left(\frac{1}{N_{\text{f}}} p_{\text{I}} + \frac{N_{\text{f}}-1}{N_{\text{f}}} p_{\text{FF}}\right) + \frac{1}{2} f\right). \quad \text{(A31)}$$

Notice that $p_{\text{female|maternal}}$ and $p_{\text{female|paternal}}$ are derived in a way analogous to $p_{\text{female|individual}}$, but conditional on picking the maternal-origin or paternal-origin gene, respectively, from the random female. Therefore, $p_{\text{female|individual}}$ is the arithmetic mean of $p_{\text{female|maternal}}$ and $p_{\text{female|paternal}}$. Analogously, $p_{\text{male|individual}}$ is the arithmetic mean of $p_{\text{male|maternal}}$ and $p_{\text{male|paternal}}$.

Substituting the appropriate consanguinities, including (A7), in the equations above yields $p_{\text{male|paternal}}$, $p_{\text{female|paternal}}$, $p_{\text{male|maternal}}$, and $p_{\text{female|maternal}}$ in their explicit form (Table A3). Dividing $p_{\text{male|paternal}}$, $p_{\text{female|paternal}}$, $p_{\text{male|maternal}}$, and $p_{\text{female|maternal}}$ by $p_{\text{I}}$ yields $R_{\text{male|paternal}}$, $R_{\text{female|paternal}}$, $R_{\text{male|maternal}}$, and $R_{\text{female|maternal}}$, respectively, in their explicit form (Table A3). Notice that $R_{\text{female|individual}}$ is the arithmetic mean of $R_{\text{female|maternal}}$ and $R_{\text{female|paternal}}$, and $R_{\text{male|individual}}$ is the arithmetic mean of $R_{\text{male|maternal}}$ and $R_{\text{male|paternal}}$.

### *5.3 Individual-based simulations for belligerence evolution*

We develop an individual-based simulation model of belligerence evolution for the purpose of illustration and to assess the robustness of our analytical results. We consider a population of $p$ = 400 groups, each containing $N_{\text{f}}$ = 10 adult females and $N_{\text{m}}$ =10 adult males. Each individual carries a belligerence locus with two alleles, each of which is represented by a real-valued number, multiple of 0.02, lying between 0 and 1, including the extremes. At initialisation, all allele values are assigned a randomly-chosen allowed value. Each adult female produces $k$ = 100 daughters and $k$ = 100 sons, mating randomly each time, so that the offspring of the same mother do not always share the same father (absolute promiscuity). When each offspring is produced, each gene mutates with probability $M$ = 0.001 to a new value, which can be the current value, the current value + 0.02, or the current value – 0.02, with equal probability. Subadult females migrate to a randomly-selected group with probability $m_{\text{f}}$; subadult males to a randomly-selected group probability $m_{\text{m}}$. Warfare is modelled as follows: for tractability, we imagine that all groups are arranged in a circle (at every generation the order of groups is randomised, to avoid neighbour effects); each group attacks the next one in the circle clockwise with a probability determined by the average belligerence allelic value of subadult males in the group; the group wins the war with probability $\omega$ = 0.5; each individual is assigned a competitiveness value in their own group ("home"), which depends on whether the group has been attacked and which group won, and a competitiveness value in the neighbouring group ("abroad"), which depends on whether the focal group has attacked that group and which group won (see Methods). During the following phase (density dependent regulation), $N_{\text{f}}$ = 10 females are randomly sampled (using competitiveness values as weights) to become adults; analogously $N_{\text{m}}$ = 10 males are randomly

sampled (using competitiveness values as weights) to become adults. These then produce the next generation of subadults in the following cycle. We track $G$ = 10,000 generations of evolution and calculate average allelic values of belligerence in each generation. The data points shown in Figure A5.3.1 come from a single simulation and are the mean of the average allelic values of the last 1000 generations of evolution. The simulation code is provided in a *Wolfram Mathematica* file, available online.

Simulation results show a reasonable fit with our analytical predictions (Fig A5.3.1), given that evolutionary trajectories in the simulation model are influenced by random drift and spontaneous mutation. Simulation results are noisier under maternal and paternal control than under individual control: this conforms to our expectation that stochastic effects would be stronger under maternal and paternal control, because in these cases selection acts on a much smaller number of individuals (i.e. mothers or fathers, rather than subadults males themselves).

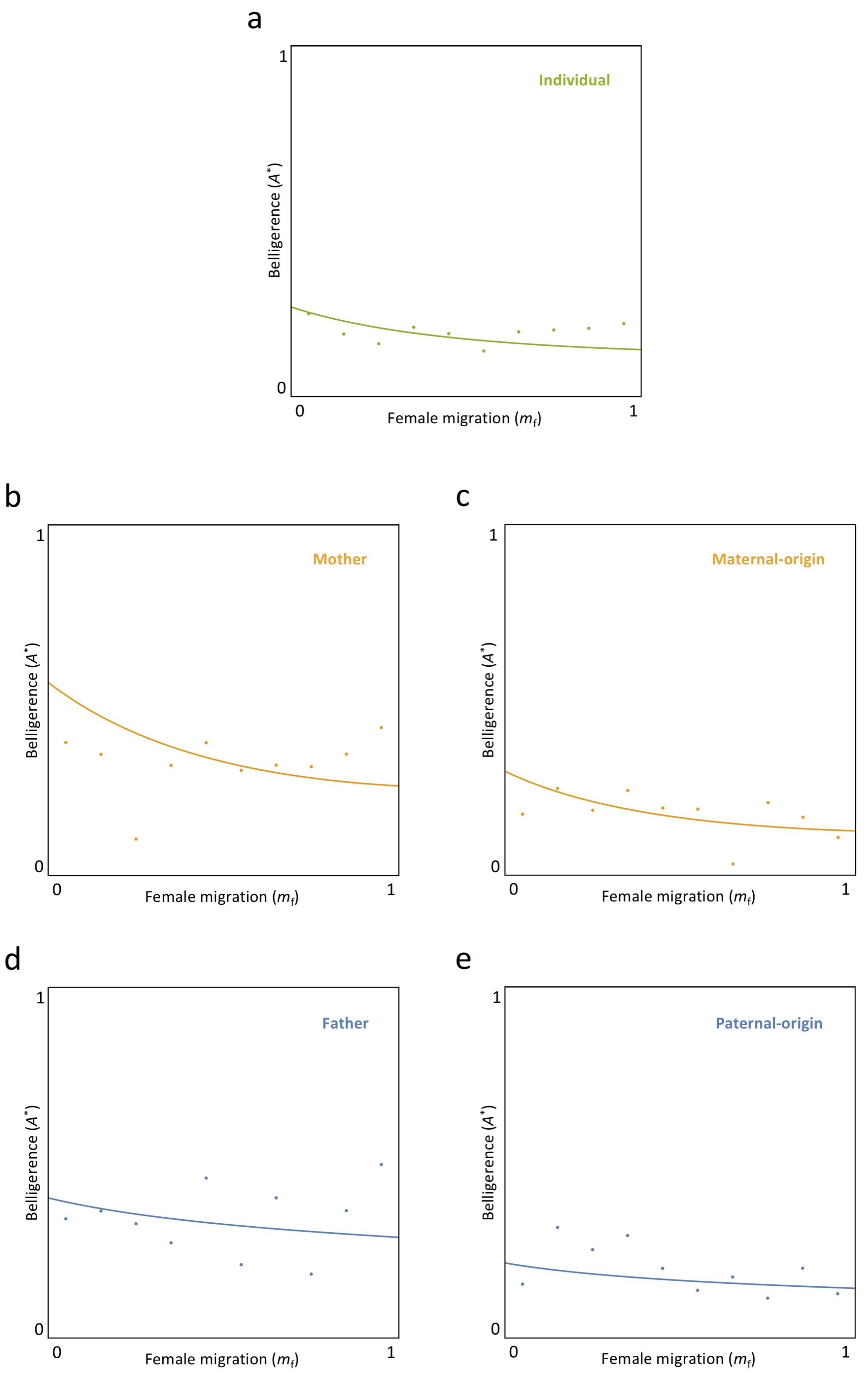


**Figure A5.3.1 – Evolution of belligerence.** Analytical predictions (lines) and individual-based simulation results (filled circles) for convergence-stable levels of belligerence ($A^*$) as a function of female migration ($m_f$) when belligerence is controlled by the focal male (panel a), his mother (panel b), his father (panel d), his maternal-origin genes (panel c), or his paternal-origin genes (panel e). Other parameter values are $\omega = 0.5$, $m_m = 0.5$, $s_f = 1$, $s_m = 0$, $N_f = N_m = 10$. We assume functional forms $a = A_{att}$ and $t = 1 - 0.025\ a^2$.

## 6 Bravery

### *6.1 Marginal fitness*

A procedure analogous to the one followed in section *5.1. Marginal fitness* is followed to study the evolution of the bravery gene. The condition for an increase in population average bravery:

$$-c_{\omega} p_{\text{focal|controller}} + \left(1 - 2\bar{\omega} s_{\text{m}} (1 - s_{\text{m}})\right) c_{\omega} p_{\text{male|controller}} + 2(1 - s_{\text{m}}) b_{\omega} p_{\text{male|controller}} + 2(1 - s_{\text{f}}) b_{\omega} p_{\text{female|controller}} > 0 \,, \tag{A32}$$

where: $c_{\omega} = -(\partial\tau(\Omega)/\partial\Omega)/\tau(\bar{\Omega})|_{\Omega=\Omega'=\bar{\Omega}}$ and $b_{\omega} = \partial\omega(\bar{\Omega}, \Omega')/\partial\Omega'|_{\Omega=\Omega'=\bar{\Omega}}$.

Condition (A32) can be rearranged as follows:

$$\frac{c_{\omega}}{b_{\omega}} < \frac{2(1-m_{\text{m}})^2\left((1-s_{\text{m}})\, p_{\text{male|controller}} + (1-s_{\text{f}})\, p_{\text{female|controller}}\right)}{(1-m_{\text{m}})^2\, p_{\text{focal|controller}} - \tilde{\varphi}_{\text{MM}}\, p_{\text{male|controller}}} \,, \tag{A33}$$

where $\tilde{\varphi}_{\text{MM}} = (1 - 2\bar{\omega} s_{\text{m}} (1 - s_{\text{m}}))(1 - m)^2$ is the probability that two males in a post-competition group were born in the same group, conditioned on the two males being involved in a war. The RHS of (A33) can be conceptualised as the "potential for bravery" (cf. [76]) and it is termed $\lambda_{\Omega}$.

Substituting $p_{\text{focal|controller}}$, $p_{\text{male|controller}}$, and $p_{\text{female|controller}}$ in (A33) with the appropriate consanguinities (see *5.2 Consanguinity and relatedness*) yields the condition for increase of the bravery gene. When the phenotype is controlled by the genotype of the father of the focal individual ($p_{\text{s}}$, $p_{\text{male|father}}$, $p_{\text{female|father}}$) this is:

$$\frac{c_{\omega}}{b_{\omega}} < \frac{2(1-m_{\text{m}})\left((1-s_{\text{m}})(1-m_{\text{m}})+(1-s_{\text{f}})(1-m_{\text{f}})\right)\left(4N_{\text{m}}N_{\text{f}} - N_{\text{f}}(N_{\text{m}}-1)\varphi_{\text{MM}} - N_{\text{m}}(N_{\text{f}}-1)\varphi_{\text{FF}} - 2N_{\text{m}}N_{\text{f}}\varphi_{\text{MF}} + (N_{\text{m}}+N_{\text{f}})\varphi_{\text{MF}} + (N_{\text{m}}+N_{\text{f}})(\varphi_{\text{MF}}+\varphi_{\text{MM}})(N_{\text{m}}-1)\right)}{(N_{\text{m}}-\tilde{\varphi}_{\text{MM}})(4N_{\text{m}}N_{\text{f}} - N_{\text{f}}(N_{\text{m}}-1)\varphi_{\text{MM}} - N_{\text{m}}(N_{\text{f}}-1)\varphi_{\text{FF}} - 2N_{\text{m}}N_{\text{f}}\varphi_{\text{MF}} + (N_{\text{m}}+N_{\text{f}})\varphi_{\text{MF}}) - \tilde{\varphi}_{\text{MM}}(N_{\text{m}}+N_{\text{f}})(\varphi_{\text{MF}}+\varphi_{\text{MM}})(N_{\text{m}}-1)} \,. \tag{A34}$$

Setting $m_{\text{m}} = m_{\text{f}} = m$ and assuming that $N_{\text{m}} = N_{\text{f}} = N$ is large, thus neglecting terms of order $1/N^2$, these reduce to:

$$\frac{c_{\omega}}{b_{\omega}} < \frac{4x(1-m)^2\left(1+\frac{1}{2}\bar{a}\,\bar{\omega}(1-m)^2(s_{\text{f}}-s_{\text{m}})(1-s_{\text{f}}-s_{\text{m}})\right)}{N\left(1-(1-m)^2\left(1-2\bar{a}\,\bar{\omega}\,x(1-x)\right)\right)} \,, \tag{A35}$$

where $x = 1 - (s_{\text{f}} + s_{\text{m}})/2$. Noting that $b_{\omega} = \bar{\omega} B_{\omega} / (1 - m_{\text{m}})$ and $c_{\omega} = C_{\omega}$ in Lehmann & Feldman's [7] notation, this recovers their result see (see Eq. (3.2) in [50]).

When the phenotype is controlled by the genotype of the mother of the focal individual ($p_{\text{s}}$, $p_{\text{male|mother}}$, $p_{\text{female|mother}}$) the conditions are given by:

$$\frac{c_{\omega}}{b_{\omega}} < \frac{2(1-m_{\text{m}})\left((1-s_{\text{m}})(1-m_{\text{m}})+(1-s_{\text{f}})(1-m_{\text{f}})\right)\left(4N_{\text{m}}N_{\text{f}} - N_{\text{f}}(N_{\text{m}}-1)\varphi_{\text{MM}} - N_{\text{m}}(N_{\text{f}}-1)\varphi_{\text{FF}} - 2N_{\text{m}}N_{\text{f}}\varphi_{\text{MF}} + (N_{\text{m}}+N_{\text{f}})\varphi_{\text{MF}} + (N_{\text{m}}+N_{\text{f}})(\varphi_{\text{MF}}+\varphi_{\text{FF}})(N_{\text{f}}-1)\right)}{(N_{\text{f}}-\tilde{\varphi}_{\text{MM}})(4N_{\text{m}}N_{\text{f}} - N_{\text{f}}(N_{\text{m}}-1)\varphi_{\text{MM}} - N_{\text{m}}(N_{\text{f}}-1)\varphi_{\text{FF}} - 2N_{\text{m}}N_{\text{f}}\varphi_{\text{MF}} + (N_{\text{m}}+N_{\text{f}})\varphi_{\text{MF}}) - \tilde{\varphi}_{\text{MM}}(N_{\text{m}}+N_{\text{f}})(\varphi_{\text{MF}}+\varphi_{\text{FF}})(N_{\text{f}}-1)} \,. \tag{A36}$$

When the genotype of the focal individual controls the phenotype ($p_{\text{I}}$, $p_{\text{male|individual}}$, $p_{\text{female|individual}}$) these is:

$$\frac{c_\omega}{b_\omega} < \frac{2(1-m_\mathrm{m})\big((1-s_\mathrm{m})(1-m_\mathrm{m})+(1-s_\mathrm{f})(1-m_\mathrm{f})\big)(N_\mathrm{f}+N_\mathrm{m})}{4N_\mathrm{m}N_\mathrm{f}-N_\mathrm{f}(N_\mathrm{m}-1)\varphi_\mathrm{MM}-N_\mathrm{m}(N_\mathrm{f}-1)\varphi_\mathrm{FF}-2N_\mathrm{m}N_\mathrm{f}\varphi_\mathrm{MF}-\tilde{\varphi}_\mathrm{MM}(N_\mathrm{m}+N_\mathrm{f})}. \quad \text{(A37)}$$

Finally, granting full control over the phenotype to paternal-origin genes ($p_{\mathrm{I|paternal}}$, $p_{\mathrm{male|paternal}}$, $p_{\mathrm{female|paternal}}$) we have:

$$\frac{c_\omega}{b_\omega} < \frac{2(1-m_m)\big((1-s_\mathrm{m})(1-m_\mathrm{m})+(1-s_\mathrm{f})(1-m_\mathrm{f})\big)(4N_\mathrm{m}N_\mathrm{f}-N_\mathrm{f}(N_\mathrm{m}-1)\varphi_\mathrm{MM}-N_\mathrm{m}(N_\mathrm{f}-1)\varphi_\mathrm{FF}+(N_\mathrm{f}+N_\mathrm{m})(N_\mathrm{m}-1)\varphi_\mathrm{MM}+N_\mathrm{m}(N_\mathrm{m}-N_\mathrm{f})\varphi_\mathrm{MF})}{N_\mathrm{m}(8N_\mathrm{f}N_\mathrm{m}-2N_\mathrm{f}(N_\mathrm{m}-1)\varphi_\mathrm{MM}-2N_\mathrm{m}(N_\mathrm{f}-1)\varphi_\mathrm{FF}-4N_\mathrm{f}N_\mathrm{m}\varphi_\mathrm{MF})-\tilde{\varphi}_\mathrm{MM}(4N_\mathrm{f}N_\mathrm{m}-N_\mathrm{f}(N_\mathrm{m}-1)\varphi_\mathrm{MM}-N_\mathrm{m}(N_\mathrm{f}-1)\varphi_\mathrm{FF}+(N_\mathrm{f}+N_\mathrm{m})(N_\mathrm{m}-1)\varphi_\mathrm{MM}+N_\mathrm{m}(N_\mathrm{m}-N_\mathrm{f})\varphi_\mathrm{MF})}, \quad \text{(A38)}$$

and granting full control over the phenotype to paternal-origin genes ($p_{\mathrm{I|maternal}}$, $p_{\mathrm{male|maternal}}$, $p_{\mathrm{female|maternal}}$), we obtain:

$$\frac{c_\omega}{b_\omega} < \frac{2(1-m_\mathrm{m})\big((1-s_\mathrm{m})(1-m_\mathrm{m})+(1-s_\mathrm{f})(1-m_\mathrm{f})\big)(4N_\mathrm{m}N_\mathrm{f}-N_\mathrm{f}(N_\mathrm{m}-1)\varphi_\mathrm{MM}-N_\mathrm{m}(N_\mathrm{f}-1)\varphi_\mathrm{FF}+(N_\mathrm{f}+N_\mathrm{m})(N_\mathrm{f}-1)\varphi_\mathrm{FF}+N_\mathrm{f}(N_\mathrm{f}-N_\mathrm{m})\varphi_\mathrm{MF})}{N_\mathrm{f}(8N_\mathrm{f}N_\mathrm{m}-2N_\mathrm{f}(N_\mathrm{m}-1)\varphi_\mathrm{MM}-2N_\mathrm{m}(N_\mathrm{f}-1)\varphi_\mathrm{FF}-4N_\mathrm{f}N_\mathrm{m}\varphi_\mathrm{MF})-\tilde{\varphi}_\mathrm{MM}(4N_\mathrm{f}N_\mathrm{m}-N_\mathrm{f}(N_\mathrm{m}-1)\varphi_\mathrm{MM}-N_\mathrm{m}(N_\mathrm{f}-1)\varphi_\mathrm{FF}+(N_\mathrm{f}+N_\mathrm{m})(N_\mathrm{f}-1)\varphi_\mathrm{FF}+N_\mathrm{f}(N_\mathrm{f}-N_\mathrm{m})\varphi_\mathrm{MF})}. \quad \text{(A39)}$$

## *6.2 Individual-based simulations for bravery evolution*

We develop an individual-based simulation model of bravery evolution for the purpose of illustration and to assess the robustness of our analytical results. We consider a population of $p$ = 400 groups, each containing $N_\mathrm{f}$ = 10 adult females and $N_\mathrm{m}$ =10 adult males. Each individual carries a bravery locus with two alleles, each of which is represented by a real-valued number, multiple of 0.02, lying between 0 and 1, including the extremes. At initialisation, all allele values are assigned a randomly-chosen allowed value. Each adult female produces $k$ = 100 daughters and $k$ = 100 sons, mating randomly each time, so that the offspring of the same mother do not always share the same father (absolute promiscuity). When each offspring is produced, each gene mutates with probability $M$ = 0.001 to a new value, which can be, the current value the current value + 0.02, or the current value – 0.02, with equal probability. Subadult females migrate to a randomly-selected group with probability $m_\mathrm{f}$; subadult males to a randomly-selected group probability $m_\mathrm{m}$. Warfare is modelled as follows: for tractability, we imagine that all groups are arranged in a circle (at every generation the order of groups is randomised, to avoid neighbour effects); each group attacks the next one in the circle clockwise with a probability equal to the optimal belligerence value under individual control, as predicted by our analytical model (see Fig. 2a); the group wins the war with probability $\omega = 0.5(1 + \Omega_\mathrm{att} - \Omega_\mathrm{def})$, where $\Omega_\mathrm{att}$ is the average bravery allelic value of subadult males in the attacking group and where $\Omega_\mathrm{def}$ is the average bravery allelic value of subadult males in the defending group; each individual is assigned a competitiveness value in their own group ("home"), which depends on whether the group has been attacked and which group won, and a competitiveness value in the neighbouring group ("abroad"), which depends on whether the focal group has attacked that group and which group won (see Methods). During the following phase (density dependent regulation), $N_\mathrm{f}$ = 10 females are randomly sampled (using competitiveness values as weights) to become adults; analogously $N_\mathrm{m}$ = 10 males are randomly sampled (using competitiveness values as weights) to become adults. These then produce the next generation of subadults in the following cycle. We

track $G$ = 10,000 generations of evolution and calculate average allelic values of bravery in each generation. The data points shown in Figure A6.3.1 come from a single simulation and are the mean of the average allelic values of the last 1000 generations of evolution. The simulation code is provided in a *Wolfram Mathematica* file, available online.

Simulation results show a reasonable fit with our analytical predictions (Fig A6.3.1), given that evolutionary trajectories in the simulation model are influenced by random drift and spontaneous mutation. Simulation results are noisier than those for belligerence (Fig A5.3.1): this conforms to our expectation that stochastic effects would be stronger for bravery than for belligerence, because bravery is expressed only when a group is involved in a war. Secondly, simulation results are noisier under maternal and paternal control than under individual control: this conforms to our expectation that stochastic effects would be stronger under maternal and paternal control, because in these cases selection acts on a much smaller number of individuals (i.e. mothers or fathers, rather than subadults males themselves).

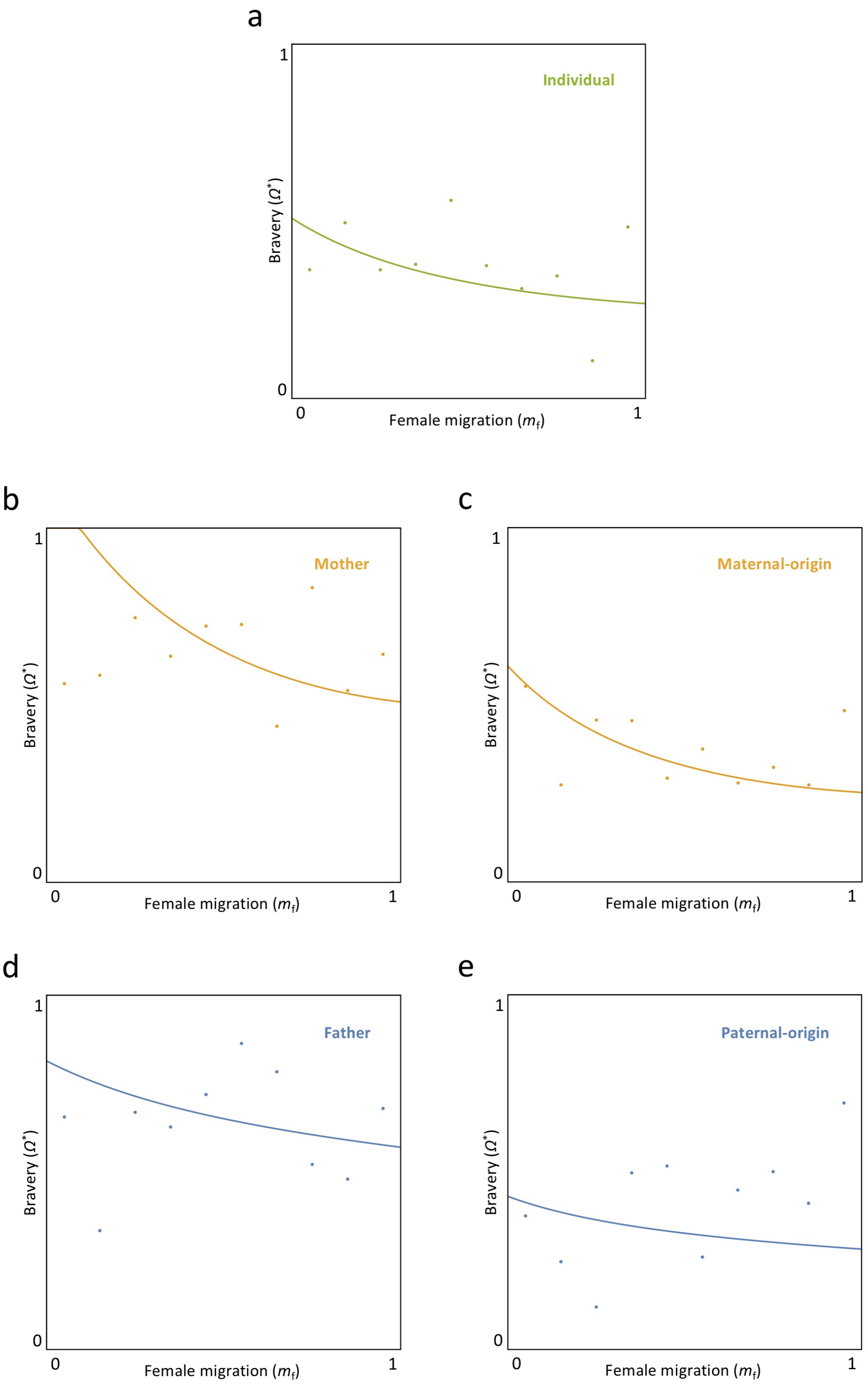


**Figure A6.3.1 – Evolution of bravery.** Analytical predictions (lines) and individual-based simulation results (filled circles) for convergence-stable levels of bravery ($\Omega^*$) as a function of female migration ($m_f$) when bravery is controlled by the focal male (panel a), his mother (panel b), his father (panel d), his maternal-origin genes (panel c), or his paternal-origin genes (panel e). Other parameter values are $\bar{a} = A^*_{ind}$, $m_m = 0.5$, $s_f = 1$, $s_m = 0$, $N_f = N_m = 10$. We assume functional forms $\omega(\Omega_{att}, \Omega_{def}) = ½ (1 + \Omega_{att} – \Omega_{def})$ and $\tau = 1 – 0.025\ \Omega^2$.

## 7 Additional references

73. Fisher RA. 1930 *The genetical theory of natural selection*. Oxford, UK: The Clarendon Press.

74. Price GR, Smith CAB. 1972 Fisher's Malthusian parameter and reproductive value. *Ann. Hum. Genet.*, **36**, 1-7. (doi:10.1111/j.1469-1809.1972.tb00577.x)

75. Bulmer M. 1994 *Theoretical evolutionary ecology.* Sunderland, MA: Sinauer Associates.

76. Gardner A. 2010 Sex-biased dispersal of adults mediates the evolution of altruism among juveniles. *J. Theor. Biol.*, **262**, 339-345. (doi:10.1016/j.jtbi.2009.09.028)

# 8 Tables

**Table A1 – Coefficients of consanguinity and relatedness under focal individual control**

| SYMBOL | VALUE |
|---|---|
| $\boldsymbol{p}_{\mathrm{I}}$ | $\frac{8\,N_{\mathrm{f}}\,N_{\mathrm{m}} - 2N_{\mathrm{f}}(N_{\mathrm{m}}-1)\,\varphi_{\mathrm{MM}} - 2N_{\mathrm{m}}(N_{\mathrm{f}}-1)\,\varphi_{\mathrm{FF}} - 4N_{\mathrm{f}}\,N_{\mathrm{m}}\,\varphi_{\mathrm{MF}}}{2(8\,N_{\mathrm{f}}\,N_{\mathrm{m}} - 2N_{\mathrm{f}}(N_{\mathrm{m}}-1)\,\varphi_{\mathrm{MM}} - 2N_{\mathrm{m}}(N_{\mathrm{f}}-1)\,\varphi_{\mathrm{FF}} - (4N_{\mathrm{f}}\,N_{\mathrm{m}} + N_{\mathrm{m}} + N_{\mathrm{f}})\varphi_{\mathrm{MF}})}$ |
| $\boldsymbol{p}_{\mathbf{male\|controller}}$ | $(1-m_{\mathrm{m}})^2 \frac{N_{\mathrm{f}} + N_{\mathrm{m}}}{8\,N_{\mathrm{f}}\,N_{\mathrm{m}} - 2N_{\mathrm{f}}(N_{\mathrm{m}}-1)\,\varphi_{\mathrm{MM}} - 2N_{\mathrm{m}}(N_{\mathrm{f}}-1)\,\varphi_{\mathrm{FF}} - (4N_{\mathrm{f}}\,N_{\mathrm{m}} + N_{\mathrm{m}} + N_{\mathrm{f}})\varphi_{\mathrm{MF}}}$ |
| $\boldsymbol{p}_{\mathbf{female\|controller}}$ | $(1-m_{\mathrm{m}})(1-m_f) \frac{N_{\mathrm{f}} + N_{\mathrm{m}}}{8\,N_{\mathrm{f}}\,N_{\mathrm{m}} - 2N_{\mathrm{f}}(N_{\mathrm{m}}-1)\,\varphi_{\mathrm{MM}} - 2N_{\mathrm{m}}(N_{\mathrm{f}}-1)\,\varphi_{\mathrm{FF}} - (4N_{\mathrm{f}}\,N_{\mathrm{m}} + N_{\mathrm{m}} + N_{\mathrm{f}})\varphi_{\mathrm{MF}}}$ |
| $\boldsymbol{R}_{\mathbf{male\|controller}}$ | $(1-m_{\mathrm{m}})^2 \frac{2(N_{\mathrm{f}} + N_{\mathrm{m}})}{8\,N_{\mathrm{f}}\,N_{\mathrm{m}} - 2N_{\mathrm{f}}(N_{\mathrm{m}}-1)\,\varphi_{\mathrm{MM}} - 2N_{\mathrm{m}}(N_{\mathrm{f}}-1)\,\varphi_{\mathrm{FF}} - 4N_{\mathrm{f}}\,N_{\mathrm{m}}\,\varphi_{\mathrm{MF}}}$ |
| $\boldsymbol{R}_{\mathbf{female\|controller}}$ | $(1-m_{\mathrm{m}})(1-m_f) \frac{2(N_{\mathrm{f}} + N_{\mathrm{m}})}{8\,N_{\mathrm{f}}\,N_{\mathrm{m}} - 2N_{\mathrm{f}}(N_{\mathrm{m}}-1)\,\varphi_{\mathrm{MM}} - 2N_{\mathrm{m}}(N_{\mathrm{f}}-1)\,\varphi_{\mathrm{FF}} - 4N_{\mathrm{f}}\,N_{\mathrm{m}}\,\varphi_{\mathrm{MF}}}$ |

**Table A2 – Coefficients of consanguinity and relatedness under mother and father control**

| SYMBOL | VALUE |
|---|---|
| $\boldsymbol{p}_{\mathrm{S}}$ | $\frac{4\,N_f\,N_m - N_f(N_m-1)\,\varphi_{MM} - N_m(N_f-1)\,\varphi_{FF} - 2N_f\,N_m\varphi_{MF} + (N_m+N_f)\varphi_{MF}}{2(8\,N_f\,N_m - 2N_f(N_m-1)\,\varphi_{MM} - 2N_m(N_f-1)\,\varphi_{FF} - (4N_f\,N_m + N_m + N_f)\varphi_{MF})}$ |
| $\boldsymbol{p}_{\mathrm{male}\vert\mathrm{father}}$ | $(1-m_m)^2\,\frac{1}{N_m}\left(\frac{4\,N_f\,N_m - N_f(N_m-1)\,\varphi_{MM} - N_m(N_f-1)\,\varphi_{FF} - 2N_mN_f\varphi_{MF} + (N_f+N_m)\varphi_{MF} + (N_m-1)(\varphi_{MF}+\varphi_{MM})(N_f+N_m)}{2(8\,N_f\,N_m - 2N_f(N_m-1)\,\varphi_{MM} - 2N_m(N_f-1)\,\varphi_{FF} - (4N_f\,N_m + N_m + N_f)\varphi_{MF})}\right)$ |
| $\boldsymbol{p}_{\mathrm{female}\vert\mathrm{father}}$ | $(1-m_m)(1-m_f)\,\frac{1}{N_m}\left(\frac{4\,N_fN_m - N_f(N_m-1)\varphi_{MM} - N_m(N_f-1)\varphi_{FF} - 2N_mN_f\varphi_{MF} + (N_f+N_m)\varphi_{MF} + (N_m-1)(\varphi_{MF}+\varphi_{MM})(N_f+N_m)}{2(8\,N_f\,N_m - 2N_f(N_m-1)\,\varphi_{MM} - 2N_m(N_f-1)\,\varphi_{FF} - (4N_f\,N_m + N_m + N_f)\varphi_{MF})}\right)$ |
| $\boldsymbol{p}_{\mathrm{male}\vert\mathrm{mother}}$ | $(1-m_m)^2\,\frac{1}{N_f}\left(\frac{4\,N_f\,N_m - N_f(N_m-1)\,\varphi_{MM} - N_m(N_f-1)\,\varphi_{FF} - 2N_mN_f\varphi_{MF} + (N_f+N_m)\varphi_{MF} + (N_f-1)(\varphi_{MF}+\varphi_{FF})(N_f+N_m)}{2(8\,N_f\,N_m - 2N_f(N_m-1)\,\varphi_{MM} - 2N_m(N_f-1)\,\varphi_{FF} - (4N_f\,N_m + N_m + N_f)\varphi_{MF})}\right)$ |
| $\boldsymbol{p}_{\mathrm{female}\vert\mathrm{mother}}$ | $(1-m_m)(1-m_f)\,\frac{1}{N_f}\left(\frac{4\,N_f\,N_m - N_f(N_m-1)\,\varphi_{MM} - N_m(N_f-1)\,\varphi_{FF} - 2N_mN_f\varphi_{MF} + (N_f+N_m)\varphi_{MF} + (N_f-1)(\varphi_{MF}+\varphi_{FF})(N_f+N_m)}{2(8\,N_f\,N_m - 2N_f(N_m-1)\,\varphi_{MM} - 2N_m(N_f-1)\,\varphi_{FF} - (4N_f\,N_m + N_m + N_f)\varphi_{MF})}\right)$ |
| $\boldsymbol{R}_{\mathrm{male}\vert\mathrm{father}}$ | $(1-m_m)^2\,\frac{1}{N_m}\left(\frac{4\,N_f\,N_m - N_f(N_m-1)\,\varphi_{MM} - N_m(N_f-1)\,\varphi_{FF} - 2N_mN_f\varphi_{MF} + (N_f+N_m)\varphi_{MF} + (N_m-1)(\varphi_{MF}+\varphi_{MM})(N_f+N_m)}{4\,N_f\,N_m - N_f(N_m-1)\,\varphi_{MM} - N_m(N_f-1)\,\varphi_{FF} - 2N_f\,N_m\varphi_{MF} + (N_m+N_f)\varphi_{MF}}\right)$ |
| $\boldsymbol{R}_{\mathrm{female}\vert\mathrm{father}}$ | $(1-m_m)(1-m_f)\,\frac{1}{N_m}\left(\frac{4\,N_fN_m - N_f(N_m-1)\varphi_{MM} - N_m(N_f-1)\varphi_{FF} - 2N_mN_f\varphi_{MF} + (N_f+N_m)\varphi_{MF} + (N_m-1)(\varphi_{MF}+\varphi_{MM})(N_f+N_m)}{4\,N_f\,N_m - N_f(N_m-1)\,\varphi_{MM} - N_m(N_f-1)\,\varphi_{FF} - 2N_f\,N_m\varphi_{MF} + (N_m+N_f)\varphi_{MF}}\right)$ |
| $\boldsymbol{R}_{\mathrm{male}\vert\mathrm{mother}}$ | $(1-m_m)^2\,\frac{1}{N_f}\left(\frac{4\,N_f\,N_m - N_f(N_m-1)\,\varphi_{MM} - N_m(N_f-1)\,\varphi_{FF} - 2N_mN_f\varphi_{MF} + (N_f+N_m)\varphi_{MF} + (N_f-1)(\varphi_{MF}+\varphi_{FF})(N_f+N_m)}{4\,N_f\,N_m - N_f(N_m-1)\,\varphi_{MM} - N_m(N_f-1)\,\varphi_{FF} - 2N_f\,N_m\varphi_{MF} + (N_m+N_f)\varphi_{MF}}\right)$ |
| $\boldsymbol{R}_{\mathrm{female}\vert\mathrm{mother}}$ | $(1-m_m)(1-m_f)\,\frac{1}{N_f}\left(\frac{4\,N_f\,N_m - N_f(N_m-1)\,\varphi_{MM} - N_m(N_f-1)\,\varphi_{FF} - 2N_mN_f\varphi_{MF} + (N_f+N_m)\varphi_{MF} + (N_f-1)(\varphi_{MF}+\varphi_{FF})(N_f+N_m)}{4\,N_f\,N_m - N_f(N_m-1)\,\varphi_{MM} - N_m(N_f-1)\,\varphi_{FF} - 2N_f\,N_m\varphi_{MF} + (N_m+N_f)\varphi_{MF}}\right)$ |

**Table A3 – Coefficients of consanguinity and relatedness under paternal- and maternal-origin genes control**

| SYMBOL | VALUE |
|---|---|
| $\boldsymbol{p}_{\mathrm{I}}, \boldsymbol{p}_{\mathrm{I}\mid\mathrm{paternal}}$ $\boldsymbol{p}_{\mathrm{I}\mid\mathrm{maternal}}$ | $\frac{8\,N_{\mathrm{f}}\,N_{\mathrm{m}} - 2N_{\mathrm{f}}(N_{\mathrm{m}}-1)\,\varphi_{\mathrm{MM}} - 2N_{\mathrm{m}}(N_{\mathrm{f}}-1)\,\varphi_{\mathrm{FF}} - 4N_{\mathrm{f}}\,N_{\mathrm{m}}\,\varphi_{\mathrm{MF}}}{2(8\,N_{\mathrm{f}}\,N_{\mathrm{m}} - 2N_{\mathrm{f}}(N_{\mathrm{m}}-1)\,\varphi_{\mathrm{MM}} - 2N_{\mathrm{m}}(N_{\mathrm{f}}-1)\,\varphi_{\mathrm{FF}} - (4N_{\mathrm{f}}\,N_{\mathrm{m}} + N_{\mathrm{m}} + N_{\mathrm{f}})\varphi_{\mathrm{MF}})}$ |
| $\boldsymbol{p}_{\mathrm{male}\mid\mathrm{paternal}}$ | $(1-m_{\mathrm{m}})^2\,\frac{1}{N_{\mathrm{m}}}\left(\frac{4N_{\mathrm{f}}\,N_{\mathrm{m}} - N_{\mathrm{f}}(N_{\mathrm{m}}-1)\,\varphi_{\mathrm{MM}} - N_{\mathrm{m}}(N_{\mathrm{f}}-1)\,\varphi_{\mathrm{FF}} + (N_{\mathrm{f}}+N_{\mathrm{m}})(N_{\mathrm{m}}-1)\varphi_{\mathrm{MM}} + N_{\mathrm{m}}(N_{\mathrm{m}}-N_{\mathrm{f}})\varphi_{\mathrm{MF}}}{2(8\,N_{\mathrm{f}}\,N_{\mathrm{m}} - 2N_{\mathrm{f}}(N_{\mathrm{m}}-1)\,\varphi_{\mathrm{MM}} - 2N_{\mathrm{m}}(N_{\mathrm{f}}-1)\,\varphi_{\mathrm{FF}} - (4N_{\mathrm{f}}\,N_{\mathrm{m}} + N_{\mathrm{m}} + N_{\mathrm{f}})\varphi_{\mathrm{MF}})}\right)$ |
| $\boldsymbol{p}_{\mathrm{female}\mid\mathrm{paternal}}$ | $(1-m_{\mathrm{m}})(1-m_{\mathrm{f}})\,\frac{1}{N_{\mathrm{m}}}\left(\frac{4N_{\mathrm{f}}\,N_{\mathrm{m}} - N_{\mathrm{f}}(N_{\mathrm{m}}-1)\,\varphi_{\mathrm{MM}} - N_{\mathrm{m}}(N_{\mathrm{f}}-1)\,\varphi_{\mathrm{FF}} + (N_{\mathrm{f}}+N_{\mathrm{m}})(N_{\mathrm{m}}-1)\varphi_{\mathrm{MM}} + N_{\mathrm{m}}(N_{\mathrm{m}}-N_{\mathrm{f}})\varphi_{\mathrm{MF}}}{2(8\,N_{\mathrm{f}}\,N_{\mathrm{m}} - 2N_{\mathrm{f}}(N_{\mathrm{m}}-1)\,\varphi_{\mathrm{MM}} - 2N_{\mathrm{m}}(N_{\mathrm{f}}-1)\,\varphi_{\mathrm{FF}} - (4N_{\mathrm{f}}\,N_{\mathrm{m}} + N_{\mathrm{m}} + N_{\mathrm{f}})\varphi_{\mathrm{MF}})}\right)$ |
| $\boldsymbol{p}_{\mathrm{male}\mid\mathrm{maternal}}$ | $(1-m_{\mathrm{m}})^2\,\frac{1}{N_{\mathrm{f}}}\left(\frac{4N_{\mathrm{f}}\,N_{\mathrm{m}} - N_{\mathrm{f}}(N_{\mathrm{m}}-1)\,\varphi_{\mathrm{MM}} - N_{\mathrm{m}}(N_{\mathrm{f}}-1)\,\varphi_{\mathrm{FF}} + (N_{\mathrm{f}}+N_{\mathrm{m}})(N_{\mathrm{f}}-1)\varphi_{\mathrm{FF}} + N_{\mathrm{f}}(N_{\mathrm{f}}-N_{\mathrm{m}})\varphi_{\mathrm{MF}}}{2(8\,N_{\mathrm{f}}\,N_{\mathrm{m}} - 2N_{\mathrm{f}}(N_{\mathrm{m}}-1)\,\varphi_{\mathrm{MM}} - 2N_{\mathrm{m}}(N_{\mathrm{f}}-1)\,\varphi_{\mathrm{FF}} - (4N_{\mathrm{f}}\,N_{\mathrm{m}} + N_{\mathrm{m}} + N_{\mathrm{f}})\varphi_{\mathrm{MF}})}\right)$ |
| $\boldsymbol{p}_{\mathrm{female}\mid\mathrm{maternal}}$ | $(1-m_{\mathrm{m}})(1-m_{\mathrm{f}})\,\frac{1}{N_{\mathrm{f}}}\left(\frac{4N_{\mathrm{f}}\,N_{\mathrm{m}} - N_{\mathrm{f}}(N_{\mathrm{m}}-1)\,\varphi_{\mathrm{MM}} - N_{\mathrm{m}}(N_{\mathrm{f}}-1)\,\varphi_{\mathrm{FF}} + (N_{\mathrm{f}}+N_{\mathrm{m}})(N_{\mathrm{f}}-1)\varphi_{\mathrm{FF}} + N_{\mathrm{f}}(N_{\mathrm{f}}-N_{\mathrm{m}})\varphi_{\mathrm{MF}}}{2(8\,N_{\mathrm{f}}\,N_{\mathrm{m}} - 2N_{\mathrm{f}}(N_{\mathrm{m}}-1)\,\varphi_{\mathrm{MM}} - 2N_{\mathrm{m}}(N_{\mathrm{f}}-1)\,\varphi_{\mathrm{FF}} - (4N_{\mathrm{f}}\,N_{\mathrm{m}} + N_{\mathrm{m}} + N_{\mathrm{f}})\varphi_{\mathrm{MF}})}\right)$ |
| $\boldsymbol{R}_{\mathrm{male}\mid\mathrm{paternal}}$ | $(1-m_{\mathrm{m}})^2\,\frac{1}{N_{\mathrm{m}}}\left(\frac{4N_{\mathrm{f}}\,N_{\mathrm{m}} - N_{\mathrm{f}}(N_{\mathrm{m}}-1)\,\varphi_{\mathrm{MM}} - N_{\mathrm{m}}(N_{\mathrm{f}}-1)\,\varphi_{\mathrm{FF}} + (N_{\mathrm{f}}+N_{\mathrm{m}})(N_{\mathrm{m}}-1)\varphi_{\mathrm{MM}} + N_{\mathrm{m}}(N_{\mathrm{m}}-N_{\mathrm{f}})\varphi_{\mathrm{MF}}}{8\,N_{\mathrm{f}}\,N_{\mathrm{m}} - 2N_{\mathrm{f}}(N_{\mathrm{m}}-1)\,\varphi_{\mathrm{MM}} - 2N_{\mathrm{m}}(N_{\mathrm{f}}-1)\,\varphi_{\mathrm{FF}} - 4N_{\mathrm{f}}\,N_{\mathrm{m}}\,\varphi_{\mathrm{MF}}}\right)$ |
| $\boldsymbol{R}_{\mathrm{female}\mid\mathrm{paternal}}$ | $(1-m_{\mathrm{m}})(1-m_{\mathrm{f}})\,\frac{1}{N_{\mathrm{m}}}\left(\frac{4N_{\mathrm{f}}\,N_{\mathrm{m}} - N_{\mathrm{f}}(N_{\mathrm{m}}-1)\,\varphi_{\mathrm{MM}} - N_{\mathrm{m}}(N_{\mathrm{f}}-1)\,\varphi_{\mathrm{FF}} + (N_{\mathrm{f}}+N_{\mathrm{m}})(N_{\mathrm{m}}-1)\varphi_{\mathrm{MM}} + N_{\mathrm{m}}(N_{\mathrm{m}}-N_{\mathrm{f}})\varphi_{\mathrm{MF}}}{8\,N_{\mathrm{f}}\,N_{\mathrm{m}} - 2N_{\mathrm{f}}(N_{\mathrm{m}}-1)\,\varphi_{\mathrm{MM}} - 2N_{\mathrm{m}}(N_{\mathrm{f}}-1)\,\varphi_{\mathrm{FF}} - 4N_{\mathrm{f}}\,N_{\mathrm{m}}\,\varphi_{\mathrm{MF}}}\right)$ |
| $\boldsymbol{R}_{\mathrm{male}\mid\mathrm{maternal}}$ | $(1-m_{\mathrm{m}})^2\,\frac{1}{N_{\mathrm{f}}}\left(\frac{4N_{\mathrm{f}}\,N_{\mathrm{m}} - N_{\mathrm{f}}(N_{\mathrm{m}}-1)\,\varphi_{\mathrm{MM}} - N_{\mathrm{m}}(N_{\mathrm{f}}-1)\,\varphi_{\mathrm{FF}} + (N_{\mathrm{f}}+N_{\mathrm{m}})(N_{\mathrm{f}}-1)\varphi_{\mathrm{FF}} + N_{\mathrm{f}}(N_{\mathrm{f}}-N_{\mathrm{m}})\varphi_{\mathrm{MF}}}{8\,N_{\mathrm{f}}\,N_{\mathrm{m}} - 2N_{\mathrm{f}}(N_{\mathrm{m}}-1)\,\varphi_{\mathrm{MM}} - 2N_{\mathrm{m}}(N_{\mathrm{f}}-1)\,\varphi_{\mathrm{FF}} - 4N_{\mathrm{f}}\,N_{\mathrm{m}}\,\varphi_{\mathrm{MF}}}\right)$ |
| $\boldsymbol{R}_{\mathrm{female}\mid\mathrm{maternal}}$ | $(1-m_{\mathrm{m}})(1-m_{\mathrm{f}})\,\frac{1}{N_{\mathrm{f}}}\left(\frac{4N_{\mathrm{f}}\,N_{\mathrm{m}} - N_{\mathrm{f}}(N_{\mathrm{m}}-1)\,\varphi_{\mathrm{MM}} - N_{\mathrm{m}}(N_{\mathrm{f}}-1)\,\varphi_{\mathrm{FF}} + (N_{\mathrm{f}}+N_{\mathrm{m}})(N_{\mathrm{f}}-1)\varphi_{\mathrm{FF}} + N_{\mathrm{f}}(N_{\mathrm{f}}-N_{\mathrm{m}})\varphi_{\mathrm{MF}}}{8\,N_{\mathrm{f}}\,N_{\mathrm{m}} - 2N_{\mathrm{f}}(N_{\mathrm{m}}-1)\,\varphi_{\mathrm{MM}} - 2N_{\mathrm{m}}(N_{\mathrm{f}}-1)\,\varphi_{\mathrm{FF}} - 4N_{\mathrm{f}}\,N_{\mathrm{m}}\,\varphi_{\mathrm{MF}}}\right)$ |
